# A multicenter double-blind, placebo-controlled randomized trial to evaluate the safety and efficacy of bovine colostrum in the treatment of severe alcoholic hepatitis (SAH)

**DOI:** 10.1186/s13063-023-07505-8

**Published:** 2023-08-11

**Authors:** S. S. Sidhu, A Dusseja, Shalimar, S Nijhawan, D Kapoor, O Goyal, H Kishore

**Affiliations:** 1 Department of Gastroenterology, Dayanand Medical College and Hospital, Ludhiana, Punjab India; 2 Department of Hepatology, Post Graduate Institute of Medical Education & Research, Chandigarh, India; 3 Department of Gastroenterology & Human Nutrition Unit, All India Institute of Medical Sciences, Delhi, India; 4 Department of Gastroenterology, Sawai Man Singh Hospital, Jaipur, India; 5 Department of Hepatology, Global Hospital, Hyderabad, India

## Abstract

**Background:**

Severe alcoholic hepatitis (SAH) is associated with high mortality. Numerous studies and meta-analysis have reported that corticosteroids reduce the 28-day mortality in SAH, but not the 6-month mortality. Therefore, newer treatments for SAH need to be studied. A pilot study from our group had recently treated ten patients with SAH with bovine colostrum (BC) [20 g thrice in a day for 8 weeks] and prednisolone. This therapy improved the biological functions and 3-month mortality. However, as more and more data showed the failure of corticosteroids to improve the 3- and 6-month mortality, especially in patients with high mDF and MELD scores, we planned this trial to study the safety and efficacy of BC (without corticosteroids) in the treatment of SAH.

**Method:**

This is a multicenter, parallel, double-blind, randomized (1:1) placebo-controlled trial, which will enroll 174 patients with SAH from 5 academic centers in the India. Patients will receive freeze-dried BC or placebo by random 1:1 allocation for 4 weeks. The primary outcome measure is survival at 3 months. The secondary outcome measures are survival at 1 month, change in mDF and MELD scores, change in endotoxin and cytokines (alpha TNF, IL6, and IL8) levels, number of episodes of sepsis [pneumonia, spontaneous bacterial peritonitis (SBP), cellulitis, urinary tract infection (UTI)] from baseline to 4 weeks.

**Discussion:**

This study will evaluate the safety and efficacy of bovine colostrum in improving the survival of patients with SAH.

**Trial registration:**

ClinicalTrials.gov NCT02473341. Prospectively registered on June 16, 2015.

**Supplementary Information:**

The online version contains supplementary material available at 10.1186/s13063-023-07505-8.

## Administrative information

Note: the numbers in curly brackets in this protocol refer to SPIRIT checklist item numbers. The order of the items has been modified to group similar items (see http://www.equator-network.org/reporting-guidelines/spirit-2013-statement-defining-standard-protocol-items-for-clinical-trials/).

**Table:** 

**Title {1}**	**A Multicentre Double Blind, Placebo Controlled Randomized Trial to Evaluate the Safety and Efficacy of Bovine Colostrum in the Treatment of Severe Alcoholic Hepatitis (SAH).**
**Trial Registration { 2a and 2b}**	ClinicalTrials.gov Identifier: NCT02473
**Protocol version {3}**	**4.0 17 October 2022 (Latest Version)**
**Funding {4}**	**Dayanand Medical College & Hospital, (DMCH) Ludhiana. DMCH had only one role in the study, besides funding: DMCH Ethical committee gave approval for the conduct of the study**
**Author Details {5a}**	Sidhu SS^1^, Dusseja A^2^, Shalimar^3^, Nijhawan S^4^, Kapoor D^5^, Goyal O^6^, Kishore H^7^ ^1^ ChiefInvestigator: Ex Professor, Department of Gastroenterology, DayanandMedicalCollege andHospital, Ludhiana,Punjab Email Id: sandeepsidhu1963@gmail.com ^2^ Professor & Head, Dept.ofHepatology,Post Graduate Institute of Medical Education &Reasearch,Chandigarh Email Id: ajayduseja@yahoo.co.in ^3^Additional Professor, Department of Gastroenterology & Human Nutrition Unit, All India Institute of Medical Sciences, Delhi Email Id:drshalimar@gmail.com ^4^Professor, Department of Gastroenterology, Sawai Man Singh Hospital, Jaipur. Email Id: dr_nijhawan@yahoo.com ^5^Consultant, Department of Hepatology, Global Hospital, Hyderabad. Email Id: dharmesh_kapoor@hotmail.com,dharmesh_kapoor@yahoo.com, ^6^Professor, Department of Gastroenterology, Dayanand Medical College and Hospital, goyalomesh@yahoo.co.in ^7^Clinical Investigator, Department of Gastroenterology, Dayanand Medical College & Hospital, Ludhiana, Email ID: harsh.kash@yahoo.co.in
**Name & Contact Information of the Trial Sponsor {5b}**	**Dayanand Medical College & Hospital, (DMCH) Ludhiana Contact Information:** Research & Development Center, DMCH,Tagore Nagar, Civil Lines, Ludhiana-141001 + 91–0161-2304282 contact@researchatdmch.com
**Role of Sponsor {5c}**	DMCH takes responsibility for the initiation, oversight, and financing of this clinical trial, but has no role to play in conducting the trial.
**Composition, roles, and responsibilities of the coordinating center, steering committee, endpoint adjudication committee, data management team, and other individuals or groups overseeing the trial {5d}**	**Sidhu SS, Goyal O, Kishore H, Department of Gastroenterology, Dayanand Medical College & Hospital, Ludhiana:** Created study protocol manual. Submitted Institutional review board (IRB) application to local IRB. Later distributed copies of IRB-approved protocol and other documents to other study sites for assistance in completing the same process at their sites. Coordinating monthly conference calls and quarterly steering committee meetings. Established data collection process.Consecutive patients diagnosed to have SAH will be then registered by the site medical team onto the trial site via *Trans European Network for clinical trials services (TENALEA),* a web-based registration and randomization system, and randomized into two groups (group A and B) to receive active drug or placebo 1:1. (Explained in detail in Sects. 16 a, b, c) Monitored data collection and submission of data from all sites: The principal means of data collection and storage from participant visits will be Electronic Data Capture (EDC) via the internet using the In Form database. Data is entered into the EDC system by site personnel. (Explained in detail in Sect. 18). Sites typically notified the coordinating center of adverse events. Responsibility for dissemination of results at the end of data collection and analysis. **Steering Committee:**Composed of the 5 Principal Investigators -Sidhu SS, Dusseja A, Shalimar, Nijhawan S, Kapoor D • To support and monitor every aspect of the research project. • Make decisions necessary for a fair conduct of the study • Resolve Issues arising out of conduct of the study • Get Status Updates on the conduct of the trial • Encourage the project personnel to ensure a timely, fair and complete study **Endpoint adjudication committee:** Composed of the 5 Principal Investigators of this study—Sidhu SS, Dusseja A, Shalimar,Nijhawan S, Kapoor D^.^ **This committee monitors** ^The hard endpoints for a patient involved in this study:^ Mortality at 3 months, 1 month • Change in mDF/MELD at 1 month • Change in Endotoxin levels at 1 month • Change in cytokines (alphaTNF,IL6&IL8 levels) at 1 month • Number of episodes of sepsis (pneumonia, SBP, cellulitis, UTI) over 1 month Data management team: Composed of the following -Sidhu SS, Dusseja A, Shalimar,Nijhawan S, Kapoor D, Goyal, O, Kishore H Will record all Primary and Secondary outcome related follow up data, via the Electronic Data Capture (EDC) using the InForm database. (Complete details in Sect. 19
**Trial Status**	Recruiting, Protocol version **4.0 17 October 2022 (Latest Version), Date of 1**^**st**^** recruitment 14/11/2017** Estimated Primary Completion Date: June 2023 Estimated Study Completion Date: August 2023
**Availability of data**	The author confirm that data supporting the finding of this study available with in the article. Raw data that support finding of this study are available from the corresponding author, upon reasonable request.
**Competing interest**	We declared that we do not have any conflict of interest in this manuscript
**Funding**	There is no trial sponsor
**Authors’ contributions**	Sidhu SS: concept or design of the article; or the acquisition, analysis. Approval of final draft; Dusseja A: concept or design of the article; or the acquisition, analysis. Approval of final draft Shalimar: concept or design of the article; or the acquisition, analysis. Approval of final draftNijhawan S: concept or design of the article; or the acquisition, analysis. Approval of final draft Kapoor D: Concept or design of the article; or the acquisition, analysis. Approval of final draft. Goyal O: concept or design of the article; or the acquisition, analysis. Approval of final draft Kishore H: concept or design of the article; or the acquisition, analysis. Approval of final draft
**Acknowledgements**	None

## Introduction

### Background and rationale {6a}

Severe alcoholic hepatitis, defined by modified Maddrey’s discriminant function (mDF) ≥ 32 and/or model for end-stage liver disease (MELD) score > 20, is associated with significant morbidity and mortality [1–3].

As per the National Institute on Alcohol Abuse and Alcoholism (NIAAA) guidelines, the 90-day mortality endpoint is preferred to the traditional 30-day mortality in light of recent trials. In addition to mortality, the other endpoints are those that reflect improvements in biological function of liver (e.g., change in mDF and MELD score) [4].

In 2015, the STOPAH study demonstrated that the use of prednisolone in patients with SAH was associated with a non-significant decrease in mortality at 28 days; however, there was no significant effect on mortality at 90-day or 1-year follow-up. This study included 1103 patients from the UK, with a mean MELD (SD) (21.2 ± 6.2). This study suggested a narrow therapeutic window (mean MELD score of 21.2 ± 6.2) in which steroids are most effective [5].

In the meta-analysis of 4 controlled trials, corticosteroid use reduces risk of death in patients of SAH, within 28 days of treatment, but not in the following 6 months. This loss of efficacy over time indicates a need for new therapeutic strategies to improve medium-term outcomes [6]. Another recent meta-analysis shows that survival in SAH has not improved over time, in spite of ongoing research for many years and many therapeutic agents have been examined for efficacy. Overall survival from SAH was 74% at 28 days, 71% at 90 days, and 56% at 180 days after admission. These results demonstrate the urgent need for better treatment in AH [7]. In 2019, a Cochrane systematic review [8] that included16 studies (from 1977 to 2015) with a total of 1884 participants concluded that corticosteroids confer no clear benefit over placebo with respect to all-cause mortality at 3 months in patients with SAH. As only one trial was at low risk of bias, it is more likely that that the trials at high risk of bias were overestimating benefits of corticosteroids and overlooking harms.

In a recent large retrospective global study (Arab Pablo et al. 2021) [9], which included 3380 patients from 53 centers in 17 countries in 4 continents, the mDF scores [median(range)] ranged from 45 (27–68) to 63 (46–90) and the MELD scores [median(range) ranged from 22 (18–29) to 25 (21–31). In an adjusted survival model, corticosteroid use decreased 30-day mortality by 41% (hazard ratio [HR] 0.59; 0.47–0.74; *p* < 0.001). The survival benefit was not sustained at 90 or 180 days.

An important limitation of this study was that only 45% completed the follow-up of 180 days. A loss to follow-up of 55% patients can severely compromise a study’s validity.

Moreover, the lower prevalence of sepsis in this study versus other contemporary studies puts a question mark on whether all the infections were included in this study or that certain septic complications have been unaccounted. In conclusion, this very large study with significant limitations confirms that corticosteroid use decreases the 30-day, but not 90- or 180-day mortality rate in patients with SAH. The highest mDF in the Western studies is [median (range)] 63 (46–90) (Arab et al.). The highest MELD score is [median (range)] 25 (21–31).

Moreover, corticosteroids are contraindicated in those with renal failure, gastrointestinal bleed, pancreatitis, and active sepsis.

In a recent Indian study, the survival rate was 22% at 90 days, and the mDf score was 77.4 (range 37–235), and MELD score was 27.5 (range 19–41). In another recent study from India, of 183 patients with SAH, the median mDF was 70 (32–320) and model for end-stage liver disease (MELD) was 26 (15–40). The 90-day survival was 56%. However, only 12% could be offered steroid therapy, due to contraindications in the remaining patient [10].

Combining four contemporary Indian studies on SAH (Singh et al. 2014 [11], Singh et al. 2018 [12], Santhosh EK et al. 2022 [13]), the maximum mDF scores were 312.4 in SAH patients (Singh et al. 2018) [12] and 472 in A—ACLF patients (Santosh et al. 2022) [13].

Combining all four above Indian studies, the maximum MELD scores were 68.5 in SAH patients (Singh et al. 2018) [12] and 85 in A—ACLF patients (Santosh et al. 2022) [13].

The inference is that SAH in Indian patients is more severe than the Western patients. As per the NIAAA, SAH patients with a MELD score of > 30 or a Maddrey discriminant function of > 60 have a very poor prognosis with a very high mortality. Hence liver support approaches or urgent transplantation should be considered, rather than pharmacotherapy. A 90-day mortality endpoint is preferred to the traditional 30-day mortality in light of recent trials.

1. Hence, in light of the NIAAA recommendations, it is clear that CS is not a therapeutic option for Indian patients with SAH as the mDF and MELD scores are very high.
2. Moreover, all recent Western studies have concluded that corticosteroid use decreases the 30-day, but not 90- or 180-day mortality rate in patients with SAH.

Once again as per the NIAAA recommendations, the 90-day mortality rate is the preferred primary outcome for studies on treatment of SAH. All four recent western studies involving treatment of SAH showed that CS have no impact on the 90-day mortality. Thus, we can say with confidence that CS are not the established standard of care, especially in the Indian context.

Hence, in light of all these western studies conducted from 2015 to 2021 and NIAAA recommendations, although our pilot study conducted in 2015 had shown that treatment with a combination of bovine colostrum (BC) and corticosteroids (CS) for 4 weeks had improved significantly the mDF level and was associated with a survival rate of 90% (9/10) at 1 month and a survival rate of 70% (7/10) at 3 months, we decided that in the current larger prospective randomized control trial (RCT) which started recruiting patients in November 2017, we would only use BC as therapeutic option versus standard medical treatment, rather than use CS as an active comparator arm which has no impact on the 90-day mortality. Also it is not unethical to avoid administration of CS inpatients with a very severe form of SAH (very high mDF > 60 & MELD scores > 30).

### Bovine colostrum (BC)

#### Composition and rationale for its treatment potential in severe alcoholic hepatitis

The BC has the following components [14]: immunological factors, namely, immunoglobins, lactoferrin, lysozyme, lactoperoxidase, microRNA, glycoconjugates, B and T lymphocytes, leukocytes, interleukins, and other polypeptide-rich prolines; growth factors nutrients.

The level of fat, protein, peptides, lactose, minerals and vitamins, growth factors, cytokines, hormones, and nucleotides in BC are the highest immediately after delivery and then begin to decline most intensely within 72 h postpartum. Many factors such as calving interval, breed, age, genetic factors, heat, and humidity, in addition to the technological processes (as given below) and hygiene conditions of production influence the quality of BC [15]. Hałasa et al. reported that the colostrum harvested 2 h after delivery had the most substantial influence on gut permeability [16].

Immunoglobulins are the most important protein of BC. Colostrum contains elevated levels of IgG, IgA, and IgM, and immunoglobulins make up 70–80% of the total protein in colostrum, which is of particular importance to the neonate, as transfer of passive immunity to the calf occurs through colostrum and not via the placenta [17].

BC contains insulin growth factor-I (IGF-1) that stimulates the growth and reconstruction of cells and tissues [18]. There has been controversy whether administration of BC supplementation results in significant increase in IGF-1 in athletes.

BC contains about 7% of fat, including omega-3 and -6 fatty acids, conjugated linoleic acid, and short-chain fatty acids. Short-chain fatty acids may be essential for improving the integrity of the intestinal inner cell membrane [19].

Another important constituent of colostrum is lactoferrin, which is a cationic iron-binding multifunctional glycoprotein belonging to the transferrin family. It is present in most milk secretions and reaches particularly high concentrations in colostrum and breast milk. The concentration of lactoferrin in colostrum is 30–100 fold higher than that in milk. Typically, the concentration of lactoferrin in colostrum ranges from 1.5 to 5 mg/mL [20].

**Fig. 1:**
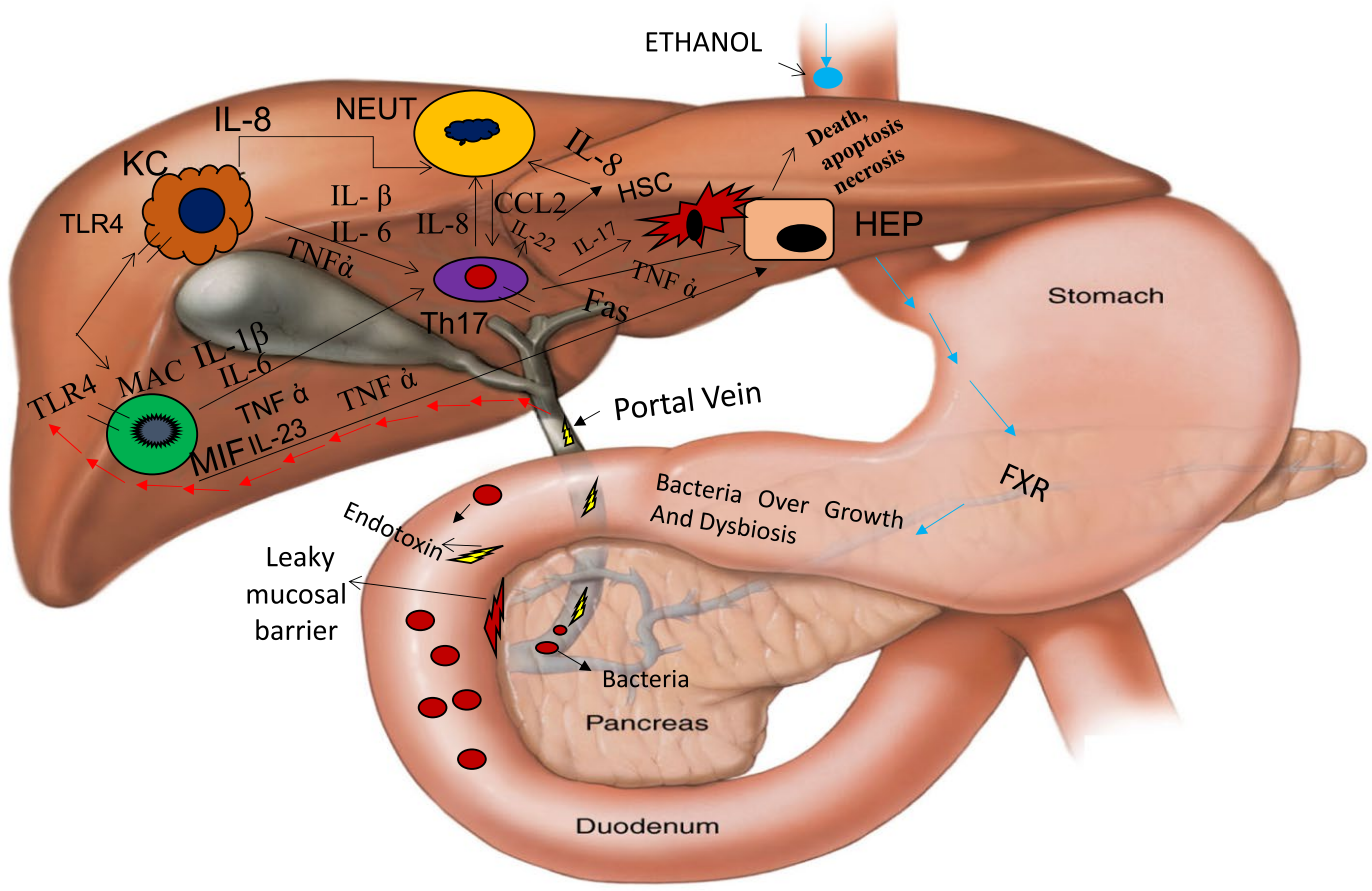
Luminal and liver action of bovine colostrum

Lactoferricin B derived from lactoferrin is bactericidal in stomach. Lactoferrin binds to lipid A of lipopolysaccharide (LPS) to neutralize it. IgG and lactoferrin synergistically neutralize lipopolysaccharide (LPS). IgG and lactoferrin interacts with mucosa-associated lymphoid tissue (MALT) of leaky mucosal barrier to convert into healthy mucosal barrier. It increases the growth and proliferation of enterocytes [21] (Fig. 1).

Additionally, lactoferrin has an impact on the levels of cytokines and chemokines that are produced by GALT cells (gut-associated lymphoid tissue) and creates an environment for the growth of beneficial bacteria in the gut [22].

#### Thermal processing, spray drying and freeze drying of colostrum

Heat treatment of milk and dairy products is aimed mainly at killing microorganisms and inactivating enzymes. Pathogens that may be transmitted to dairy calves in colostrum include Mycobacterium avium subsp. paratuberculosis, Salmonella spp., Mycoplasma spp., Listeria monocytogenes, Campylobacter spp., Mycobacterium bovis, and Escherichia coli.

It was reported that heating of colostrum to 60 °C for 120 min was sufficient to reduce the level of viable Mycoplasma bovis, Listeria monocytogenes, Escherichia coli O157:H7, Salmonella enteritidis, and Mycobacterium avium subspecies, paratuberculosis below detectable limits [23]. Similarly, in a study conducted by Donahue et al., heating colostrum at 60 °C for 60 min decreased total plate counts and coliform counts, and did not affect native IgG concentration [24]. This was associated with increased efficiency of IgG absorption and, consequently, higher serum IgG concentration in calves fed heat-treated (60 °C for 60 min) compared to raw colostrum. The precise reason for this remains unclear, but it was hypothesized that the presence of bacteria in colostrum could interfere with systemic absorption of IgG molecules in the small intestine.

These results are consistent with study conducted by Godden et al. [25] who, in addition, reported that calves fed heat-treated (60 °C for 60 min) colostrum were at significantly lower risk for any illness in the preweaning period compared with calves untreated with colostrum.

BC supplements have proven useful in the management of gastrointestinal diseases such as acute infectious diarrhea, Helicobacter pylori infections, irritable bowel syndrome, inflammatory bowel disease (IBD), and different types of human cancer cell lines (e.g., esophagus, colorectal, lung, breast, and ovarian cancer [26–28].

Bovine colostrum is used in the treatment of HIV-positive patients with chronic diarrhea [29] and rotavirus diarrhea in children [30].

Bovine colostrum supplementation at 60 g/day for 8 weeks has been shown to improve repeat sprint performance [31], peak vertical jump power and peak cycle power, and peak running speed during a repeated out of intense exercise [32].

#### Luminal and liver action of bovine colostrum

Bovine colostrum (BC) has two important components, i.e., lactoferrin (LF) and immunoglobulin G (IgG). Lactoferricin B derived from lactoferrin is bactericidal in stomach. Lactoferrin binds to lipid A of lipopolysaccharide (LPS) to neutralize it. IgG and LF synergistically neutralize LPS. IgG interacts with MALT of leaky mucosal barrier to convert into healthy mucosal barrier (HMB). Chronic ethanol exposure sensitizes Kupffer cells to activation by LPS through Toll-like receptors (TLR-4). Due to the luminal actions of BC, less bacteria, LPS (endotoxin), and *Candida albicans*-derived β-glucans—pathogen-associated molecular particles (PAMPs) enter the portal vein resulting in decreased trafficking and interaction of bacteria and LPS with TLR-4 receptors of the macrophages and Kupffer cells in the liver. This culminates in the markedly reduced production of proinflammatory cytokines such as interleukin-1 (IL-1) and tumor necrosis factor-alpha. This dampens the network of intercellular signaling between hepatic macrophages, Kupffer cells, and the heathy hepatocytes. This also results in decreased infiltration of neutrophils, monocytes, and T cells into the liver. Hepatocyte death by necrosis or apoptosis is prevented leading to lack of release of hepatocyte-derived intracellular proteins or nucleic acids—damage-associated molecular patterns (DAMPs). The hepatic stellate cell activation by cytokines such as tumor necrosis factor-alpha and resultant liver fibrosis is prevented. Thus, BC effectively induces reversal of the liver pathophysiology seen in SAH.

#### Derivation of dose of bovine colostrum

Oral consumption of bovine colostrum (Lactobin®) reduces perioperative endotoxemia and prevents reduction of endotoxin-neutralizing capacity, suggesting a stabilization of gut barrier during abdominal surgery [33].

Oral bovine colostrum preparation (Lactobin®) 56 g/day was given prophylactically to 20 patients for 3 days preoperatively and (Placebo) standard milk 56 g/day was given for 3 days, to 20 similar patients. The daily dose of Lactobin or placebo was divided into 4 parts (before breakfast, at lunch, at supper, and at night).

In the above study, major gastric surgeries were performed on six patients in each group; pancreatic surgery was done in 13 patients in the Lactobin group and 14 patients in the placebo group. The clinical course was monitored by daily determination of the APACHE-II score. The course of the plasma endotoxin (LPS) levels and the endotoxin neutralization capacity (ENC) were measured daily up to the 10th postoperative day [33]. The results showed that the LPS levels in the Lactobin group, expressed as AUC, were significantly lower than those in the control group (*p* < 0.05). The difference between the two groups was apparent on the day of the operation and the day after. The increase in endotoxin-neutralizing capacity (ENC) was significantly greater in the patients treated with the Lactobin than in the control group (*p* < 0.006). Thus, BC has been successfully used to significantly decrease the level of endotoxemia [lipopolysaccharides (LPS)].

In the second placebo-controlled randomized study [34], 60 patients who underwent coronary bypass operations were evenly enrolled. The patients were treated by enteral application of either 42 g of a bovine colostrum milk preparation per day or placebo, for 2 days preoperatively. Endotoxin and ENC were sequentially determined intra- and postoperatively. Interleukin-6, CRP, transferrin, alpha-2-macroglobulin, albumin, apo-A, apo-B, IgG, IgA, and IgM were determined by ELISA and nephelometrically. The clinical course was followed up by daily evaluation of the Apache-II score. No differences of the Apache-II score (colostrum group: 6.5 ± 1.9 vs. controls: 6.8 ± 1.8 on admission) were observed. Endotoxin levels were elevated at the end of the operation. There was no reduction in endotoxin levels and no increase in ENC levels in patients receiving the colostrum milk preparation throughout the observation period. Bovine colostrum failed to curtail perioperative endotoxemia probably because the amount of colostrum preparation administered was less. Thus, the correct dose of bovine colostrum appears to be 60 g / day [34].

Hence, the dose of bovine colostrum that was used in the present study was 60 g/day in three divided doses, i.e., 20 g (dissolved in 100 mL of water) thrice a day given for 4 weeks. The total protein content of the BC used in our study has 52.75% (527.5 mg/1 g) of the bovine colostrum powder and the IgG content was 34.25% of the total protein in the bovine colostrum powder, i.e., 180 mg of IgG/1 g of bovine colostrum powder versus 800 mg of protein/g and 52 mg of IgG/g of Lactobin® bovine colostrum. Thus, the current colostrum has a much higher content of IgG (180 mg/g of colostrum) than the Lactobin colostrum used by Bolke E et al. [34]. This is probably because of the technological advances in the preparation of bovine colostrum since 1990 in general, and more specifically because that the Spray dried technique used in Lactobin preparation decreases the IgG much more than the freeze-dried technique used in the preparation of bovine colostrum used in the current study.

### Explanation for choice of comparators {6b}

#### Experimental arm (Group A)

As discussed at length in the “Introduction,” we decided that in the current study, we would only use BC as therapeutic option versus standard medical treatment

BC is a freeze-dried powder.

#### Placebo comparator arm (Group B): pasteurized milk powder

Both the BC and the pasteurized milk powder will be of the same color, appearance, and taste.

The dose of BC has been derived from the use of oral BC preparation (Lactobin®) and standard milk powder as placebo in the study by Bolke E et al. [34].

### Objectives {7}

The purpose of this clinical research study is to study the safety and efficacy of bovine colostrum in the treatment of severe alcoholic hepatitis.

### Trial design {8}

This is multicenter, parallel, double-blind, randomized (1:1), placebo-controlled trial. This is a superiority trial where the hypotheses is that the treatment intervention (bovine colostrum) is superior to control (placebo) in the treatment of SAH.

#### Study frame work

The framework of a trial refers to its overall objective to test the superiority, non-inferiority, or equivalence of one intervention with another, or in the case of exploratory pilot trials, to gather preliminary information on the intervention (e.g., harms, pharmacokinetics) and the feasibility of conducting a full-scale trial.

## Methods: participants, inclusion, and exclusion criteria and interventions

### Study setting {9}

The study will recruit patients admitted to hospitals with SAH at five academic centers in the India. A list of participating study sites has been included in the Annexure 4.

### Eligibility criteria {10}

#### Inclusion criteria

- Age 18–75 years
- Onset of jaundice within the prior 3 months
- Ongoing consumption of > 40 g (female) or > 60 (males) g alcohol/day for 6 months or more, with less than 60 days of abstinence before the onset of jaundice
- Aspartate aminotransferase > 50
- Aspartate aminotransferase/alanine amino transferase ratio > 1.5, and both values < 400 IU/L
- Serum bilirubin (total) > 3.0 mg/dL
- Liver biopsy confirmation in patients with confounding factors—including possible ischemic hepatitis (e.g., severe upper gastrointestinal bleed, hypotension) or cocaine use within 7 days; possible drug-induced liver injury; uncertain alcohol use assessment (e.g., patient denies excessive alcohol use); and atypical laboratory tests (e.g., AST < 50 IU/mL or > 400 IU/mL, AST/ALT ratio < 1.5), antinuclear antibody > 1:160 or SMA > 1:80
- Patient with controlled upper GI bleed, resolved sepsis, and treated acute kidney injury can be enrolled
- Maddrey’s discriminant function ≥ 32 assuming a control prothrombin time of 12 s
- Model for end-stage liver disease score > 20

#### Exclusion criteria

- Jaundice more than 3 months
- AST > 500 U/L or ALT > 300 U/L (not compatible with alcoholic hepatitis)
- Other concomitant causes of liver disease: viral hepatitis, autoimmune liver disease, metabolic liver disease, vascular liver disease
- HIV positive
- Cow milk allergy or severe lactose intolerance
- Active gastrointestinal bleeding
- Uncontrolled sepsis with multi-organ failure
- Acute kidney injury at time of randomization with creatinine > 2.5 mg/dL
- Evidence of acute pancreatitis or biliary obstruction
- Subjects who are pregnant or lactating
- Significant systemic cardio-pulmonary illness
- Patients in shock, requiring the use of vasopressors or inotropic support in 12 h prior to randomization
- Treatment for alcoholic hepatitis within 1 month of study entry with corticosteroid use > 1 week.
- Any patient who has received any investigational drug or device within 30 days of entering into the study.

All baseline assessments and eligibility criteria will be implemented before randomization.

## Experimental intervention: cow colostrum powder—a lyophilized (freeze-dried) colostrum (Annexure 1)

The herds of (Kankrej Cow breed) cows are kept under close supervision in good state of hygiene without exposure to antibiotics, pesticides, and anti-helminthics.

Only the milk of the first 12 h, when the nutrient concentration has its maximum value, is used. After this milk is milked from the mother cow, it is moved directly to the collection center in a stainless steel jar by authorized vendor of colostrum milk supplier in a refrigerated van with temperature control to maintain the quality of raw milk.

After weighing the milk and verification of weight/volume, it is subjected for quality check. Then filtration of milk is followed by continuous, high-temperature short-time (HTST) pasteurization at 71.7 °C for 15–20 s in a closed vessel. The pasteurized milk is stored in tray chiller freezer for 08 h at minus 30 °C. After completion of this process, the powder is packaged as per specifications. Testing is performed of freeze-dried bovine colostrum as per specification to ensure the consistency of powder.

Thus the steps of production of bovine colostrum are outlined below:

(1) Raw colostrum moved inward. (2) Quality check. (3) Storage in freezer. (4). Pasteurization process. (5). Storage in tray chiller freezer minus ( −) 30 °C (freeze drying), (6) Finished product quality check. (7) Finished products storage.

## Strategies to improve adherence to interventions

### Study intervention {11a}

#### Experimental arm (Group A): bovine colostrum

Bovine colostrum as a freeze-dried powder 20 g (dissolved in 100 ml of water) thrice a day will be given for 4 weeks.

### Side effects of bovine colostrum: {11b} allergy

#### Placebo comparator (Group B): pasteurized milk powder

Pasteurized milk powder 20 g (dissolved in 100 ml of water) will be given thrice a day for 4 weeks.

### Side effects of pasteurized milk powder: allergy

Both the bovine colostrum and the pasteurized milk powder will be of the same color, appearance, and taste.

### Criteria for discontinuing or modifying allocated interventions for a given trial participant {11b}

#### Occurrence of adverse effects of bovine colostrum (experimental arm) and pasteurized milk powder (placebo comparator): allergy

##### Permitted study drug adjustments

Study drug has to be taken in line with the treatment and dosage prescribed in the study. Any study drug adjustments can only be made by the Principal Investigator if as per his/her judgment, this will positively impact patient treatment. Further, any deviation from the laid out treatment in the study protocol will have to be documented giving justifications thereof.

### Strategies to improve adherence to intervention protocols, and any procedures for monitoring adherence (e.g., drug tablet return, laboratory tests) {11c}

All patients will be initially admitted in the hospital ICU / wards; hence, adherence to interventional protocol will be the responsibility of the Principal Investigator. After the patient is discharged from the hospital, the patients shall return for an outpatient visit. Participants will be contacted and reminded by telephone prior to the day of the outpatient visit at 7 days, 14 days, 21 days, 28 days, and then bimonthly for 2 months. On the outpatient visit, a history and physical examination of the patient will be conducted and details noted. The patients or their caregiver will return the empty sachets (and counted) and new batch of sachets will be given till the duration of the next outpatient visit. This process shall continue till the completion of 28 days of the study. Thereafter, the patients will be telephonically reminded to return for history, physical examination, and performance of laboratory tests till 90 days.

### Relevant concomitant care and interventions that are permitted or prohibited during the trial {11d}

Standard of care treatment will be given in both arms which are as follows:

1. Oral + enteral nutrition (if oral intake is insufficient: protein 1.2–1.5 g/kg/day, energy (kcal) 35–40/day, B complex vitamins daily. [35]
2. Nutrition education with dietitian including implementation of night-time snacks [36]
3. Assess vitamin D levels in alcoholic hepatitis with underlying cirrhosis, as deficiency of vitamin D is highly prevalent. Supplement vitamin D orally with serum vitamin D levels < 20 ng/ml, to reach serum vitamin D (25(OH)D) > 30 ng/ml. In patients with alcoholic hepatitis with underlying cirrhosis and ascites, sodium restriction (recommended intake of sodium 80 mmol day = 2 g of sodium corresponding to 5 g of salt added daily to the diet according to disease) taking care to ease mildly sodium restriction to improve diet palatability [35].
4. Antibiotics for sepsis; diuretics for ascites; lactulose / rifaximin / L ornithine L aspartate for hepatic encephalopathy; terlipressin, and albumin for type 1 hepatorenal syndrome; carvedilol for prophylaxis against variceal hemorrhage; terlipressin with endoscopic variceal ligation for variceal bleed, if indicated.
5. Other concomitant medication

All medications (other than study drugs) and significant non-drug therapies (including physical therapy and blood transfusion) administered after the patient starts treatment with study drug must be listed on the Concomitant Medications/Significant Non-drug Therapies in the record file.

### Investigations (Annexure 2)

Each patient shall have an:

- Abdominal ultrasonography
- Upper gastrointestinal endoscopy would be done, if indicated.
- Liver biopsy: Percutaneous *or* transjugular liver biopsies may be obtained before start of therapy for diagnosis of alcoholic hepatitis. *Liver biopsy confirmation is needed in patients with confounding factors.*
- Laboratory tests: Hemogram, prothrombin time, blood glucose, liver function tests, blood urea, serum creatinine, and serum electrolytes will be done at baseline, and at 7 days, 14 days, 21 days, 28 days, and then bimonthly for 2 months or *earlier, if indicated.*
- Microbiologic tests: Blood cultures, urine culture, cultures of aspirates from endotracheal tubes in ventilated patients for aerobic and anaerobic bacteria, and fungi shall be done at admission. A diagnostic paracentesis will be done in all patients with ascites, at baseline, to diagnose spontaneous bacterial peritonitis. A repeat cell count (total and differential) shall be done on day 5 in patients diagnosed to have spontaneous bacterial peritonitis.
- A chest radiograph shall be done.
- Cytokine levels: Endotoxin, alphaTNF, and IL6&IL8 levels will be done at baseline and at end of treatment.
- Tests for etiologic evaluation

Etiology of hepatitis will be taken as *alcohol* if there is a history of significant alcohol intake (average consumption of more than three drinks (~40 g) per day for women and four drinks (~50–60 g) per day for men) for 6 months or more, with less than 60 days of abstinence before the onset of jaundice.

Each patient will be tested for:

- Hepatitis B surface antigen (HBsAg) and anti-hepatitis C (HCV) antibody using a third-generation commercial ELISA will be done for all patients.
- Wherever indicated, autoimmune hepatitis will be diagnosed when compatible clinical signs and symptoms, laboratory abnormalities (serum AST or ALT, and increased serum total IgG), serological (ANA, SMA, anti-LKM 1, or anti-LC1), and histological (interface hepatitis) findings are present.
- Wherever indicated, hemochromatosis will be diagnosed using serum iron, TIBC, transferrin saturation, and ferritin levels.

Wilson disease as suggested by liver disease seen in young patients (< 40 years) with

(A) Cirrhosis with chronic hepatitis, or
(B) Acute hepatitis with liver failure (females–males being ratio 4:1) with severe jaundice, a slight elevation of the activity of transaminases, low alkaline phosphatase, and low hemoglobin concentrations (Coombs-negative hemolytic anemia) and acute renal failure.

- Wilson’s disease diagnoses shall be confirmed only in these patients by the presence of Kayser-Fleischer rings, a low-serum caeruloplasmin concentration (less than 0.20 g/dl), and a raised 24-h urine copper excretion. In untreated symptomatic patients, “baseline” copper excretion greater than > 1.6 μmol/24 h (> 100 μg/24 h) is taken as diagnostic of Wilson’s disease. Hepatic copper concentration (on liver biopsy) ≥ 250 μg/g dry weight (considered diagnostic); < 50 μg/g almost always excludes diagnosis)

i. In addition, pregnancy test for females of childbearing potential will be done at baseline

### Outcomes {12}

#### Primary outcome measure

Survival at 3 months.

#### Secondary outcome measure

- Survival at 1 month
- Change in mDF/MELD at 1 month
- Change in endotoxin levels at 1 month
- Change in cytokines (alphaTNF, IL6&IL8 levels) at 1 month
- Number of episodes of sepsis (pneumonia, SBP, cellulitis, UTI) over 1 month

### Participant timeline {13}

**Fig. 2:**
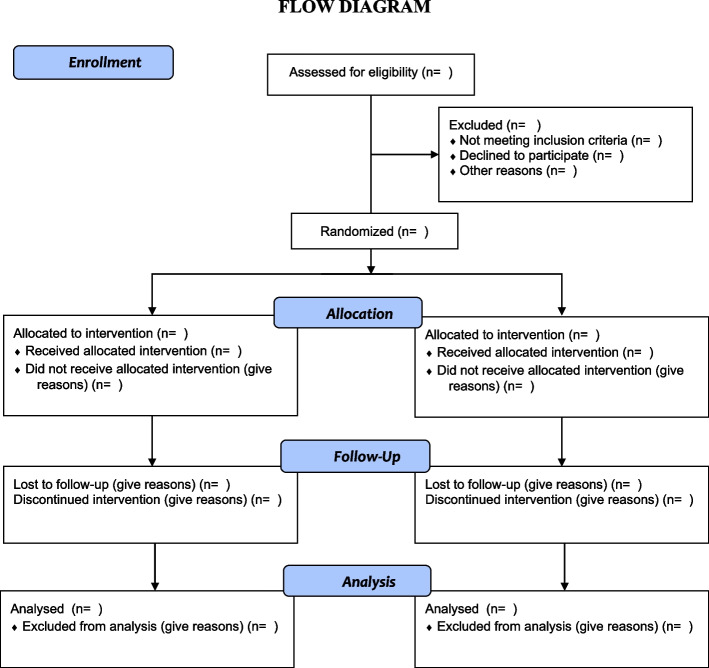
Flow diagram

Time schedule of enrolment, interventions (including any run-ins and washouts), assessments, and visits for participants. A schematic diagram is highly recommended (Fig. 2).

### Sample size determination {14}

In a recent Indian study, published in 2014 by Singh et al. [11] the mDf score was 77.4 (range 37–235), MELD score was 27.5 (range 19–41), and the survival rate was 22% at 90 days.

Hence, we calculated the sample size on the basis of the above study.

We consider a 20% increase in survival rate (80% power at 5% alpha) to be clinically meaningful and sufficient to change practice. To detect a 20% increase in survival (from 22 to 42%) in 3 months [the sample size was based on survival at a fixed time point (22% vs 42% at 3 months)], we require 79 patients in each group. Assuming 10% dropouts, the total sample size required for the study is 174.

### Recruitment strategies for achieving adequate participant enrolment to reach target sample size {15}

Regular Principal and Clinical Investigator meetings will be held to support trial recruitment and evaluate any recruitment challenges.

## Methods: assignment of interventions

### Allocation

#### Sequence generation {16a}

Enrolment of patients, assessing eligibility, recording of all baseline parameters, and obtaining informed consent will be carried out by Principal Investigator. Consecutive patients diagnosed to have SAH will be then registered by the site medical team onto the trial site via *Trans European Network for clinical trials services (TENALEA)*, a web-based registration and randomization system, and randomized into two groups (group A and B) to receive active drug or placebo 1:1. Non-eligible patients will be deemed screening failures.

#### Allocation concealment mechanism {16b}

Subsequently the TENALEA registration system will release the randomization code to non-study personnel in every center. Treatment allocation will be blinded to the patient or care giver or to the Principal Investigator by providing each patient with a unique number from the TENALEA registration system.

#### Implementation {16c}

The randomization code will be sent to non-study personnel in every center. These personnel will prepare closed envelopes with printed randomization numbers along with the corresponding code (A or B) for the treatment group.

Each center will have serially numbered identical sachets containing bovine colostrum or pasteurized bovine milk managed and administered to the patients by non-study personnel according to the random sequence. The drug and the placebo will be identical in color, taste, and appearance to ensure masking. The study drug will be administered by an oral route. The Principal Investigator and his team will have no information about the group allocation.

#### Blinding (masking) {17a}

The treatment arm will be concealed to patients, investigators (Clinicians), data collectors, outcome assessors, data analysts, and statistician. The randomization code will be revealed to the study statistician only after the completion of recruitment, data collection, and data analysis has been carried out.

#### Procedure for unblinding when necessary {17 b}

Unblinding may be permitted in the event of a medical emergency where breaking the blind is required to provide medical care to the subject.

Local Principal Investigators (PI) will have access to a mechanism that permits rapid unblinding should they feel this is necessary. *Local Standard Operating Procedures* describing the emergency unblinding procedure will be in place. The chief investigator recommends, but does not require, that the investigator contact him before breaking the blind. The rationale for unblinding must be clearly explained in source documentation and on the electronic case report form (eCRF), along with the date on which the treatment assignment information was obtained.

## Methods: data collection, management, and analysis

### Data collection methods {18a}

The Principal Investigator and his clinical trial team members will.

- Collect the primary and secondary outcome-related data: Baseline data (including USG abdomen and UGI endoscopy)
- Collect follow-up data, from participant visits. Duplicate measurements of all data will be collected.
- Obtain prior medications list and evaluate current medications.
- Collect blood for laboratory tests as per Annexure 2.

The principal means of data collection and storage from participant visits, permitted alteration to the intervention protocol, adherence to the interventions while admitted in the hospital, patient follow-up as outpatient visit, recording outpatient history and physical exam and laboratory data will be Electronic Data Capture (EDC) via the internet using the InForm database. Data is entered into the EDC system by site personnel. All source data recorded in the electronic case report form (eCRF) will be signed by the Investigator or his/her appropriate designee. All changes made following the electronic signing will have an electronic audit trail with a signature and date. Specific instructions and further details will be outlined in the eCRF manual.

### Data management {18 b}

The clinical trial team shall closely follow-up every patient at baseline (day 1), 4, 7, 21, 28, 45, 60, 75, and 90 days and remind him / her telephonically to attend all their scheduled outpatient visits till the end of follow-up at 3 months The team shall make a record of all outcome data collected for participants who discontinue or deviate from intervention protocols.

At each outpatient visit, medical history (together with a disease-specific history related to the patient’s diagnosis of severe alcoholic hepatitis) is taken and complete physical examination is performed. Primary and secondary outcome-related follow-up data, from participant visits, shall be accurately collected, and duplicate measurements of all data will be collected and recorded via the Electronic Data Capture (EDC) using the InForm database. All laboratory tests as mentioned in Annexure 2 will be performed and the laboratory test values recorded via the Electronic Data Capture (EDC) using the In Form database. The patient will be queried for adverse events, adherence to study medication, and intake of any concurrent medications and recorded in the In Form database.

### Data analysis {18c}

#### Statistical analysis

The Principal Investigator will assess the quality of the collected data by studying the InForm database collected by Electronic Data Capture (EDC). This includes Primary and Secondary outcome-related data including laboratory tests as per Annexure 2: baseline and follow-up data, from participant visits including record of all outcome data collected for participants who discontinue or deviate from intervention protocols.

### Data management {19}

The Principal Investigator will assess the quality of the collected data. All the data recorded in the electronic case report form (eCRF) will be signed by the Investigator or his/her appropriate designee. All changes made following the electronic signing will have an electronic audit trail with a signature and date. Specific instructions and further details will be outlined in the eCRF manual. The responsibility of coding, security, and storage of all data is with the *Trans European Network for clinical trials services (TENALEA).*

### Statistical analysis {20a}

#### Statistical methods

##### Statistical methods for primary and secondary outcomes {20a}

Subject population (S) for analysis

Common sensitivity analyses will be attempted to assess the robustness of the results to protocol deviations. For primary survival (efficacy) analysis, we will use intention to treat analysis, in such a way that all study subjects who are randomized, regardless of adherence to study medication, will be used in the analysis in the treatment arms to which they are originally assigned to at randomization (“as randomized”). Thus, the patient set used for the primary analysis according to the ITT principle is called “full analysis set”.

As a supplementary analysis, we will perform a per-protocol analysis using patients who will achieve at least 80% compliance (without any major protocol violations) with treatment arms. Significance will be assessed using a two-sided alpha level of 0.05.

#### Statistical methods

The baseline patients’ characteristics mentioned in Annexure 2 including comorbid-conditions will be compared between the two treatment groups to demonstrate the actual study group balance and to ensure whether a proper randomization is established or not. We will measure central tendency and variability with means and standard deviations or medians and interquartile range for continuous characteristics and frequencies with percentages for categorical characteristics.

For primary outcome “survival at 3 months,” we will first use an unadjusted log-rank test of the Kaplan–Meier survival estimates for the two treatment arms at a two-sided alpha level of 0.05 with survival censored at 3 months. Further, a Cox proportional hazards regression model (with survival censored at 3 months) will be used to estimate the hazards ratio (HR) with 95% confidence interval (CI) associated with treatment arm (A) compared to the reference treatment (B). Parameters which are observed as unbalanced between the groups will be adjusted in the above model. We will also add any potential time-dependent covariates to the model, such as AST and ALT levels at months 1, 2, and 3 to estimate their predictive value for mortality.

Similar unadjusted log-rank test of the Kaplan–Meier survival estimates, and Cox proportional hazards regression model analysis will be planned for secondary outcome survival at 1 month. Other secondary outcomes measures such as change in mDF levels/MELD score, endotoxin levels, and cytokines levels between baseline and 4-week assessment will be assessed for normal distribution by graphical (Q–Q Plot) interpretation and numerical method (Kolmogorov–Smirnov test). A paired *t*-test will be used for all those outcomes which will follow approximately normal distribution and a sign-rank test will be used for skewed parameters. Proportionality assumption will be assessed for the considered ordinal outcome number of episodes of sepsis (pneumonia, SBP, cellulitis, and UTI). Partial proportional odds ratio regression will be used to assess the effect of treatment on the number of episodes of sepsis (pneumonia, SBP, cellulitis, UTI) over 4 weeks. A proportional partial odds model will be used if the treatment group variable will not meet the proportional odds assumption and will have different effects on different severity levels of sepsis.

#### Item 20b

##### Additional analysis

Subgroup analysis may be conducted based on the clinical age group criteria and grams of alcohol consumed criteria. The imbalanced baseline characteristics will not be included in the primary analysis; however, we will conduct additional exploratory analysis where such characteristics will be included to assess the robustness of the primary analysis.

#### Item 20c

##### Subject population (S) for analysis

Common sensitivity analyses will be attempted to assess the robustness of the results to protocol deviations. For primary survival (efficacy) analysis, we will use intention to treat analysis, in such a way that all study subjects who are randomized, regardless of adherence to study medication, will be used in the analysis in the experimental arms to which they are originally allocated. As a supplementary analysis, we will perform a per-protocol analysis using patients who will achieve at least 80% compliance (without any major protocol violations) with experimental arms. Significance will be assessed using a two-sided alpha level of 0.05.

#### Analysis of missing data

It is important to document and investigate the reasons for loss to follow-up. We will examine the distribution of baseline characteristics between patients who completed the treatment with those who dropped out within each treatment group to identify the known factors associated with the poor treatment compliance.

We will use Rubin’s multiple-imputation method to deal with the missing data. Although the multiple-imputation method is developed based on the missing at random (MAR) assumption, this method can handle both missing completely at random (MCAR) and missing not at random (MNAR).

Another approach is a tipping point analysis, a multiple imputations technique under MNAR assumption and will be used to impute the missing data not at random. Under this analysis, a tipping point can be defined as the difference of means for continuous data and the difference of event numbers for binary data where the *p*-values get changed. This method does not require assessing the missing data mechanism and does not involve model uncertainty and assumptions.

#### Interim analyses

No formal interim analysis is planned for this trial.

## Methods: monitoring

### Composition of the data monitoring committee, its role and reporting structure

#### Data will be monitored by the Principal Investigator of each site {21a}

##### Monitoring frequency and agenda

Monitoring will be conducted at 4, 7, 21, 28, 45, 60, 75, and 90 days and relevant information will be shared by the Principal Investigator to DMEC. During each visit, Principal Investigator will discuss with DMEC all relevant issues pertaining to the trial, validate the CRFs with the source data, and identify and modify in case of any discrepancies or errors.

##### Source data verification

The source data verification and review of records is necessary to minimize any errors in transcribing data onto the CRFs. The monitor (PI) will validate the CRF entries with the source data and subjects’ records.

##### Data clarification / rectification

Data will be entered into the CRF by site personnel. Post data entry, source data verification will be completed by Study monitor (PI). If any query requires clarification from site, data management personnel will answer the query. If the response for raised query is satisfactory, then data management personnel will close the query or if response for raised query is not satisfactory then data management personnel will collect all query related data related to site to rectify any error that may have occurred.

##### Close-out meeting / site closure

At this visit, left over trial medication(s) and trial related materials will be collected. An accounting will be done of all materials supplied to the site, as well as all patients screened and enrolled.

### Description of any interim analyses and stopping guidelines, including who will have access to these interim results and make the final decision to terminate the trial {21b}

There will be no interim analysis.

#### Study drug discontinuation / stopping guidelines

After the patient has been enrolled into the study, his/her treatment will be discontinued by the Principal Investigator if one or more of the following pertain(s):

- Occurrence of adverse effects of bovine colostrum (Group A) and pasteurized milk powder (Group B): allergy or lactose intolerance
- Treatment with prohibited concomitant medications, for example corticosteroids.

Patients who discontinue the study before completion of it will be scheduled for an end of study visit as soon as possible, at which time all of the assessments listed for the final visit will be performed.

At a minimum, all patients who discontinue the study, including those who refuse to return for a final visit will be contacted telephonically for the final visit clinical assessment:

- Improvement / worsening / death / started taking treatment from another hospital
- Safety evaluations during the 30 days following the last dose of drug administration.

### {22} Harms

Plans for collecting, assessing, reporting, and managing solicited and spontaneously reported adverse events and other unintended effects of trial interventions or trial conduct.

#### Monitoring for adverse events

A treatment-emergent adverse event (AE) will be defined as an AE that begins or that worsens in severity after at least one dose of study drug has been administered. Any adverse event (AE) occurring during the study will be documented in the subject’s case report form (CRF) specifying the time of onset, the duration, the severity, and the relationship to the test medication.

Grades of Severity of the AE as per the Common Terminology Criteria for Adverse Events (CTCAE) v 5.0:

- Grade 1 Mild AE
- Grade 2 Moderate AE
- Grade 3 Severe AE
- Grade 4 Life-threatening or disabling AE
- Grade 5 Death related to AE

### Auditing {23}

Frequency and procedures for auditing trial conduct, if any, and whether the process will be independent from investigators.

This clinical trial will be audited by the Institutional Ethical Committee (IEC) to evaluate trial conduct and compliance with the protocol, standard operating procedure, and International Conference on Harmonization Good Clinical Practice (ICH-GCP) guidelines. The PI and his team will supply all the information regarding the trial conduct to the IEC. The frequency of the audit will be at 4, 7, 21, 28, 45, 60, 75, and 90 days of the trial.

## Ethics and dissemination

### {24} Plans for seeking research ethics committee/institutional review board (REC/IRB) approval

The study protocol, patient information sheet, and informed consent form (the latter two were created in three languages—Punjabi, Hindi, English) (Annexure No. 3) were submitted to the IEC of each site for approval.

Similarly, at all other trial sites, the respective IEC’s will give approval following which the study will be initiated at the respective sites.

### {25} Plans for communicating important protocol modifications (e.g., changes to eligibility criteria, outcomes, analyses) to relevant parties (e.g., every trial site Principal Investigator, IEC, trial participant, trial registry, journal, regulator)

Any modification in the research protocol final version or Statistical Analysis Plan will be submitted to all the different trial site Principal Investigators and their IECs. These changes will be modified on the ClinicalTrials.gov site.

### 26 a. Who will obtain informed consent or assent from potential trial participants or authorized surrogates, and how (see Item 32)

Informed Consent is documented by means of a written, signed, and dated informed consent form (in the language the patient reads and or writes: Punjabi, Hindi, English). The trial patients shall voluntarily confirm his / her willingness to participate in this trial, after having been informed of all aspects of the trial that are relevant to the subject’s decision to participate. In case of the patient’s inability to give the consent because of hepatic encephalopathy, *a legally acceptable representative*, usually an immediate relative, will give consent, on behalf of a prospective patient, to the subject’s participation in the clinical trial. If the patient / relative cannot read or write, then the PI will explain in a vernacular language all aspects of the trial including the risks and benefits of a medical intervention, which in this study is bovine colostrum or milk and obtain the consent easily as most subjects are familiar with these interventions (bovine colostrum or milk).

### Who will take informed consent?

The consent of the patients will be taken by the patient’s treating physician—Principal Investigator (PI)—after explaining the benefits and risks of participating in the study. The patient will be encouraged to ask questions regarding the study. They will be given a patient information sheet (PIS) and will be given 24 h to consider the study (or less if the patient feels that he / she has decided to participate in the study) and whether to participate, prior to giving their informed consent. Patients will be given a copy of the signed informed consent form (ICF).

Potential patients for the trial who present with hepatic encephalopathy may be unable to consent for themselves but are not excluded from the trial. An informed consent was taken from the nearest relative / legal representative of the patient for enrolment in the trial, until the patient recovered his mental faculty, at which point the patient was informed about the trial and asked to decide whether or not they wanted to continue in the study.

### Additional consent provisions for collection and use of participant data and biological specimens

Informed consent forms will include the option to consent for the collection, and use of, participant data and biological specimens in ancillary studies. These include analyses of serum biomarkers of disease.

### {26b}. Additional consent provisions for collection and use of participant data and biological specimens in ancillary studies, if applicable

NA

### {27}. How personal information about potential and enrolled participants will be collected, shared, and maintained in order to protect confidentiality before, during, and after the trial

It is the duty of physicians who are involved in medical research to protect the life, health, dignity, integrity, privacy, and confidentiality of personal information of research subjects.

*Every precaution will be taken to maintain the confidentiality of their personal information.* Subjects must be identified only by their assigned identification number and initials on all CRFs and other records and documents.

### {28}. Financial and other competing interests for Principal Investigators for the overall trial and each study site

The PIs certify to their absence of financial and other competing interests. The PIs have no relationship with the company that manufactures BC which is the treatment arm in this study.

### {29}. Statement of who will have access to the final trial dataset, and disclosure of contractual agreements that limit such access for investigators

All Principal Investigators and site study personnel will have access to final trial dataset. There are no contractual agreements.

### {30}. Provisions, if any, for ancillary and post-trial care, and for compensation to those who suffer harm from trial participation

The patients will be given standard medical treatment after termination of the study. No compensation needs to be given as the experimental product is a food supplement.

## Dissemination policy

### {31} a Plans for investigators to communicate trial results to participants, healthcare professionals, the public, and other relevant groups (e.g., via publication, reporting in results databases, or other data sharing arrangements), including any publication restrictions

We, as Principal Investigators conducting this clinical trial, have registered this clinical trial is registered at ClinicalTrials.gov—ClinicalTrials.gov Identifier: NCT02473. The summary of results information will be submitted to ClinicalTrials.gov for public posting. Disseminating this information will help to advance the translation of research results into knowledge, products, and procedures that improve human health in patients with SAH. Results information from this trial will be submitted not later than 1 year after the trial’s primary completion date.

The completed study shall be submitted for publication in an appropriate medical journal. Negative and inconclusive as well as positive results will be published. Institutional affiliations will be declared in the publication.

### {31b}. Authorship eligibility guidelines and any intended use of professional writers

Authors will be selected based on the authorship criteria mentioned by International committee of medical journal editors. The corresponding author takes primary responsibility for communication with the journal during the manuscript submission, peer review, and publication process. This author ensures that all the journal’s administrative requirements, such as providing details of authorship, ethics committee approval, clinical trial registration documentation, and disclosures of relationships are reported

### {31c} Plans, if any, for granting public access to the full protocol, participant-level dataset, and statistical code

Publication of the study protocol in a journal—Trials.com (Open Access)—and publication of the entire study which incorporates participant-level dataset, and statistical code, in an appropriate medical journal will be done.

## Appendices

### {32}. Model consent form and other related documentation given to participants and authorized surrogates

Informed consent form, patient information sheet (In Punjabi, Hindi, English) are mentioned as Annexures.

### {33}. Plans for collection, laboratory evaluation, and storage of biological specimens for genetic or molecular analysis in the current trial and for future use in ancillary studies, if applicable

We plan to collect stool samples from SAH patients for subsequent analysis. Animal studies have suggested that BC significantly increases anti-inflammatory microbial commensal bacteria, such as Lachnospiraceae, Prevotellaceae, Ruminococcaceae, Akkermansiaceae, the *Eubacterium* xylanophilum group, and *Lactococcus*. On the other hand, administration of BC seems to significantly reduce various proinflammatory bacterial species, such as *Deferribacterota*, *Desulfobacterota*, *Tyzzerella*, *Peptococcus*, and *Enterococcaceae*.

### Supplementary Information

**Additional file 1: Annexure 1.** Technological process flow chart of production of Bovine Colostrum. **Annexure 2.** Visit. Schedule and assessments. **Annexure 3.** Patient information sheet (English Version). **Annexure 4.** Patient consent form (in English). **Annexure 5.** List of study sites participating in this study. **Annexure 6.** Patient information sheet (Hindi & Punjabi Version). Patient consent form (Hindi & Punjabi Version).

## Data Availability

The author confirm that data supporting the finding of this study available with in the article. Raw data that support finding of this study are available from the corresponding author, upon reasonable request.
