# Supplementary material for: A multicenter double-blind, placebo-controlled randomized trial to evaluate the safety and efficacy of bovine colostrum in the treatment of severe alcoholic hepatitis (SAH)
Source: Trials. 2023 Aug 11;24:515. doi: 10.1186/s13063-023-07505-8 (PMC10416362; doi:10.1186/s13063-023-07505-8)
Supplement: Supplementary file 1 — Additional file 1: Annexure 1. Technological process flow chart of production of Bovine Colostrum. Annexure 2. Visit. Schedule and assessments. Annexure 3. Patient information sheet (English Version). Annexure 4. Patient consent form (in English). Annexure 5. List of study sites participating in this study. Annexure 6. Patient information sheet (Hindi & Punjabi Version). Patient consent form (Hindi & Punjabi Version). [file 13063_2023_7505_MOESM1_ESM.zip › Consent Form English (2)R2.pdf]

PATIENT INFORMATION SHEET

Date:

INVESTIGATOR :______________________________
(Principal and Co-Investigator) :______________________________

Name of Participant: ___________________________

Full Title: Comparison of Bovine colostrums versus Placebo: Randomized Double blind controlled Trial in treatment of severe alcoholic hepatitis.

You are invited to take part in this research study. The information in this document is meant to help you decide whether or not to take part. Please feel free to ask if you have any queries or concerns.

You are being asked to participate in this study being conducted in DMC and Hospital, Ludhiana, Punjab, because you satisfy our eligibility criteria which are: diagnosis of Severe Alcoholic Hepatitis, age above 18 years, no contraindication to the use of the agents to be used in the study, which means absence of known allergy to bovine colostrum.

You will be one of the 250 patients we plan to recruit in this study. You will be given oral Pasteurized Bovine colostrum as a freeze dried powder or placebo (20 gm thrice a day) for 4 weeks.

What is the purpose of research?

Severe alcoholic hepatitis is associated with a very high mortality (30-40%) in first month improvement in survival with the existing therapy like glucocorticoids and pentoxiphylline is less then desired. The number of patients needed to treat to prevent one death is 5. Hence we need a better therapy to improve the survival rate without increasing the side effects.

The study design

All patients in the study will be given oral Pasteurized Bovine colostrum as a freeze dried powder or placebo (20 gm thrice a day) for 4 weeks

Study Procedures

The study involves evaluation of the effect of oral bovine colostrum in improving survival rate and mDF score. Once a patient is enrolled in the study, he/she is required to follow the instructions like taking the study drugs as instructed, taking diet as instructed. Samples for LFT will be taken at baseline, and 7th day,14 days, 21 days , 28 days and then bimonthly for two months or earlier if indicated. Other routine investigations will be done when indicated.

Women of childbearing potential
A patient must not participate if she is pregnant or breastfeeding a child.

Possible risks to you
The study drug oral bovine colostrum are have so far, in earlier studies, not demonstrated side effects except in patients with milk allergy and lactose intolerance.

Possible benefits to you
You are not expected to get any benefit from being on this research study, other than the treatment benefit.

Possible benefits to other people
The results of the research may provide benefits to the society in terms of advancement of medical knowledge and therapeutic benefit to future patients.

The alternatives you have
If you do not wish to participate, you have the alternative of getting the standard treatment for your condition.

Cost to the participant
You will not be paid to participate in this research study. In case of any adverse event occurring due to the study medications, you will be provided free treatment at our Institute and proper referral if necessary.

What should you do in case of injury or a medical problem during this research study?
Your safety is the prime concern of the research. If you have a medical problem as a result of being in this study, you should contact one of the people listed at the end of the consent form. You will be provided the required care/treatment. You will be entitled to your legal rights besides this.

Confidentiality of the information obtained from you
You have the right to confidentiality regarding the privacy of your medical information (personal details, results of physical examinations, investigations, and your medical history). By signing this document, you will be allowing the research team investigators, other study personnel, institutional ethics committee and any person or agency required by law like the Drug Controller General of India to view your data, if required.
The results of clinical tests and therapy performed as part of this research may be included in your medical record. The information from this study, if published in scientific journals or presented at scientific meetings, will not reveal your identity.

How will your decision to not participate in the study affect you?
Your decision not to participate in this research study will not affect your medical care or your relationship with the investigator or the institution. Your doctor will still take care of you and you

will not lose any benefits to which you are entitled.

Can you decide to stop participating in the study once you start?
The participation in this research is purely voluntary and you have the right to withdraw from this study at any time during the course of the study without giving any reasons. However, it is advisable that you talk to the research team prior to stopping the treatment. Though advisable that you give the investigators the reason for withdrawing, it is not mandatory.

Can the investigator take you off the study?
You may be taken off the study without your consent if you do not follow instructions of the investigators or the research team or if the investigator thinks that further participation may cause you harm.

Right to new information
If the research team gets any new information during this research study that may affect your decision to continue participating in the study, or may raise some doubts, you will be told about that information.

Contact persons
For further information / questions, you can contact us at the following address:

Principal Investigator:
Dr Sandeep Singh Sidhu/ Ph: 9814025085
Dept. of Gastroenterology
DMC and Hospital, Ludhiana Fax: 0161-2302620
email: sandeepsidhu1963@gmail.com

Co-Investigator
Dr. Omesh Goyal/ Ph: 9914821155
Dept. of Gastroenterology
DMC and Hospital, Ludhiana Fax: 0161-2302620
Email: goyalomeah@yahoo.co.in

In case of conflicts, you can contact the **convener** of our institutional ethics committee at the following address:
Dr Gagandeep Singh
Convener, Institutional Ethics Committee
DMC and Hospital
Telephone: 9815500720

Patient consent form

Title of the study: Comparison of Bovine colostrums versus Placebo: Randomized Double blind controlled Trial in treatment of severe alcoholic hepatitis.

Name of the participant: ____
Name of the Principal/Co- investigator: ________________________________
Name of the Institution: DMC and Hospital, Ludhiana

Documentation of the informed consent

I, … … … … … … … … … ., have read the information in this form (or it has been read to me). I was free to ask any questions and they have been answered. I am over 18 years of age and, exercising my free power of choice, hereby give my consent to be included as a participant in the study entitled- Comparison of Bovine colostrums versus Placebo: Randomized Double blind controlled Trial in treatment of severe alcoholic hepatitis.

(1) I have read and understood this consent form and the information provided to me.
(2) I have had the consent document explained to me.
(3) I have been explained about the nature of the study.
(4) My rights and responsibilities have been explained to me by the investigator.
(5) I have been advised about the risks associated with my participation in the study.
(6) I have informed the investigator of all the treatments I am taking or have taken in the past *6 weeks* including any *desi* (alternative) treatments.
(7) I agree to cooperate with the investigator and I will inform him immediately if I suffer unusual symptoms.
(8) I have not participated in any research study within the past 6 months.
(9) I am aware of the fact that I can opt out of the study at any time without having to give any reason and this will not affect my future treatment in the hospital.
(10) I am also aware that the investigators may terminate my participation in the study at any time, for any reason, without my consent.
(11) I hereby give permission to the investigators to release the information obtained from me as result of participation in this study to the regulatory authorities, Government agencies, and ethics committee. I understand that they may inspect my original records.
(13) My identity will be kept confidential if my data are publicly presented.
(14) If, despite following the instructions, I am physically harmed because of any substance or any procedure as stipulated in the study plan, my treatment will be carried out free at the Investigational site / the sponsor will bear all the expenses, if they are not covered by my Insurance agency or by a Government program or any third party.
(15) I have had my questions answered to my satisfaction.
(16) I have decided to be in the research study.

I am aware, that if I have any questions during this study, I should contact at one of the addresses listed above. By signing this consent from, I attest that the information given in this document has been clearly explained to me and apparently understood by me. I will be given a copy of this

consent document.

Name and signature / thumb impression of the participant
(or legal representative if participant incompetent):

_________________ (Name) ___________ (Signature)

Date: __________ Time: ________

Name and signature of impartial witness (required for illiterate patients):

__________________ (Name) ___________ (Signature)

Date:__________ Time: _________

Address and contact number of the impartial witness: ___________________
____

Name and signature of the Investigator or his representative obtaining consent:

____________________ (Name) _________________ (Signature)

____________________ (Date)

Investigator's Certificate

I certify that all the elements including the nature, purpose and possible risks of the above study as described in this consent document have been fully explained to the subject. In my judgment, the participant/legal representative possesses the legal capacity to give informed consent to participate in this research and is voluntarily and knowingly giving informed consent to participate.

Signature of the Investigator: ________________ Dated:__________

Name of the Investigator: ___________________
