# Supplementary material for: A multicenter double-blind, placebo-controlled randomized trial to evaluate the safety and efficacy of bovine colostrum in the treatment of severe alcoholic hepatitis (SAH)
Source: Trials. 2023 Aug 11;24:515. doi: 10.1186/s13063-023-07505-8 (PMC10416362; doi:10.1186/s13063-023-07505-8)
Supplement: Supplementary file 1 — Additional file 1: Annexure 1. Technological process flow chart of production of Bovine Colostrum. Annexure 2. Visit. Schedule and assessments. Annexure 3. Patient information sheet (English Version). Annexure 4. Patient consent form (in English). Annexure 5. List of study sites participating in this study. Annexure 6. Patient information sheet (Hindi & Punjabi Version). Patient consent form (Hindi & Punjabi Version). [file 13063_2023_7505_MOESM1_ESM.zip › Consent Form Hindi (2)R2.pdf]

<u>रोगी सूचना शीट</u>

दिनांक:

अन्वेषक: ____

(प्रधानएवं सह-अन्वेषक) : ________________________________

प्रतिभागी का नाम:_____________________________

पूर्ण शीर्षक: बोवेन कोलोस्ट्रम बनाम प्लेसबो की तुलना: गंभीर शराब संबंधी हेपेटाइटिस के उपचार में डबल अज्ञात प्लेसबो (प्रयोगिक औषधि) का क्रमरहित नियंत्रित परीक्षण।

इस शोध अध्ययन में भाग लेने के लिए आप आमंत्रित हैं । इस दस्तावेज़ में उपलब्ध जानकारी आप को यह तय करने में मदद करेगी कि आप इसमें भाग लें अथवा नहीं । कृपया, आप किसी भी प्रश्न या चिंता के विषय में पूछने के लिए स्वयं को स्वतंत्र अनुभव करें । आप से डीएमसी और अस्पताल, लुधियाना, पंजाब, द्वारा आयोजितअध्ययन में भाग लेने का अनुरोध किया जा रहा है क्योंकि आप हमारी पात्रता के मानदंड को पूरा करते हैं: गंभीर शराबी हैपेटाइटिस का निदान, 18 वर्ष से अधिक उम्र, अध्ययन में प्रयोग किये जाने वाले एजेंटों के उपयोग करने के लिए कोई अन्तर्विरोध नहीं, अर्थात बोवाइन (गोजातीय) कोलोस्ट्रम से एलर्जी की अनुपस्थिति है।

आप 250 रोगियों में से एक होंगे, जिन्हें इस अध्ययन में भर्ती करने की हमारी योजना है । आप को मौखिक रूप से 28 दिनों के लिए बोवाइन कोलोस्ट्रम (20 ग्राम एक दिन में तीन बार) अथवा प्लेसबो (20 ग्राम एक दिन में तीन बार) दिया जायेगा।

अनुसंधान का उद्देश्य क्या है?

गंभीर मदिरा हैपेटाइटिस पहले महीने में ही उच्च मृत्यु दर (30-40%) से जुड़ा हुआ है । ग्लुकोकोर्टिकोएड और पेंटोक्सीफाईलिन की मौजूदा चिकित्सा के साथ जीवित रहने की सम्भावना में सुधार पहले महीने में वांछित से कम है । एक मृत्यु को रोकने के लिए इलाज के लिए 5-7 रोगियों की आवश्यकता है । अतः हमें दुष्प्रभाव में वृद्धि के बिना जीवित रहने की दर में सुधार करने के लिए एक बेहतर चिकित्सा की आवश्यकता है।

अध्ययन की रूप-रेखा

अध्ययन में सभी रोगियों को मौखिक रूप से 28 दिनों के लिए बोवाइन कोलोस्ट्रम (20 ग्राम एक दिन में तीन बार) अथवा प्लेसबो (20 ग्राम एक दिन में तीन बार) दिया जायेगा ।

अध्ययन प्रक्रियायें

इस अध्ययन में 1 और 3 महीने में जीवित रहने की दर में सुधार लाने में मौखिक बोवाइन कोलोस्ट्रम के प्रभाव का मूल्यांकन सम्मिलित है । एक रोगी को एक बार अध्ययन में नियुक्त होने पर, निर्देशानुसार अध्ययन ड्रग्स लेने के निर्देशों का पालन करने की आवश्यकता है और निर्देशित आहार लेना आवश्यक है । रक्त के नमूनों को आधार-रेखा पर और 7, 14, 28, 60, 90 वें दिन पर लिया जाएगा । अन्य सामान्य जांचें संकेतानुसार की जाएँगी।

प्रसव क्षमता की महिला

एक महिला को इसमें भाग नहीं लेना चाहिए, यदि वह गर्भवती है अथवा बच्चे को स्तनपान करा रही है ।

आप के लिए संभावित खतरे

बोवाइन कोलोस्ट्रम ने अब तक, पहले के अध्ययनों में, दूध की एलर्जी और लैक्टोज असहिष्णुता को छोड़कर रोगियों में और किसी दुष्प्रभाव का प्रदर्शन नहीं किया है ।

आप के लिए संभावित लाभ

इस शोध अध्ययन में सम्मिलित होने से आप उपचार लाभ के अतिरिक्त कोई अन्य लाभ प्राप्त करने की उम्मीद नहीं कर रहे हैं ।

अन्य लोगों के लिए संभव लाभ

शोध के परिणाम भविष्य में समाज में रोगियों को चिकित्सीय ज्ञान और चिकित्सीय लाभप्रदान कर सकता है ।

आपके पास विकल्प

यदि आप भाग लेने के इच्छुक नहीं हैं, तो आप के पास अपनी अवस्था के लिए मानक उपचार लेने का विकल्प है ।

भाग लेने के लिए लागत

इस शोध अध्ययन में भाग लेने के लिए कोई भी भुगतान नहीं किया जाएगा । अध्ययन दवाओं की वजह से होने वाली किसी भी प्रतिकूल घटना के मामले में आपको हमारे संस्थान में मुफ्त इलाज की सुविधा प्रदान की जाएगी और आवश्यक होने पर उचित रेफरल प्रदान किया जाएगा ।

आप को इस शोध अध्ययन के दौरान चोट लगने पर अथवा चिकित्सीय समस्या के मामले में क्या करना चाहिए?

आपकी सुरक्षा अनुसंधान के लिए प्रमुख चिंता का विषय है ।यदि इस अध्ययन में होने के परिणामस्वरुप आप को चिकित्सीय समस्या है, तो आप को सहमति पत्र के अंत में सूचीबद्ध लोगों में से किसी एक को संपर्क करना चाहिए । आप को आवश्यक देखभाल / उपचार उपलब्ध कराया जाएगा । आप इस के अतिरिक्त अपने कानूनी अधिकारों के हकदार भी रहेंगे ।

आप से प्राप्त जानकारी की गोपनीयता

आप को अपनी चिकित्सा जानकारी (व्यक्तिगत जानकारी, शारीरिक परीक्षा, जांच के परिणाम, और अपनी चिकित्सा के इतिहास) के बारे में गोपनीयता का अधिकार है । इस दस्तावेज़ पर हस्ताक्षर करके, आप, अनुसंधान टीम के जांचकर्ताओं, अन्य अध्ययन कर्मियों, संस्थागत नैतिकता समिति और यदि आवश्यक हो, तो भारत के औषधि महानियंत्रक की तरह कानून द्वारा अपेक्षित किसी भी व्यक्ति या एजेंसी को अपने डेटा देखने की अनुमति देते हैं ।

इस शोध के भाग के रूप में किये गए नैदानिक परीक्षण और चिकित्सा के परिणाम को आपके मेडिकल रिकॉर्ड में शामिल किया जा सकता है । यदि, इस अध्ययन से प्राप्त जानकारी, वैज्ञानिक पत्रिकाओं में प्रकाशित की जाएगी अथवा वैज्ञानिक बैठकों में प्रस्तुत की जाएगी, तो आपकी पहचान उजागर नहीं की जाएगी ।

कैसे अध्ययन में भाग नहीं लेने का आपका निर्णय आप को प्रभावित करेगा?

इस शोध अध्ययन में भाग न लेने का आपका निर्णय आपकी चिकित्सीय देखभाल अथवा अन्वेषक या संस्था के साथ अपने रिश्ते को प्रभावित नहीं करेगा। अभी भी आपका डॉक्टर आप का ख्याल रखेगा और आप किसी भी लाभ को खो नहीं देंगे, जिसके आप हकदार हैं ।

एक बार अध्ययन आरम्भ करने के पश्चात् क्या आप उसमें भागीदारी रोकना तय कर सकते हैं?

इस शोध में भागीदारी पूरी तरह स्वैच्छिक है और आपको कोई भी कारण दिए बिना अध्ययन के दौरान किसी भी समय इस अध्ययन को छोड़ने का अधिकार है । हालांकि, आप को यह परामर्श दिया जाता है कि उपचार रोकने से पूर्व अनुसंधान दल से बात कर लें । आपको जांचकर्ताओं को नाम वापस लेने का कारण देने की सलाह दी जाती है, परन्तु यह अनिवार्य नहीं है ।

क्या अन्वेषक आपको अध्ययन से हटा सकता है?

आप को आपकी सहमति के बिना अध्ययन से दूर किया जा सकता है, यदि आप जांचकर्ताओं या शोध टीम के निर्देशों का पालन नहीं करते हैं अथवा अन्वेषक सोचता है कि आगे की भागीदारी से आपको नुकसान हो सकता है ।

नई जानकारी का अधिकार

यदि अनुसंधान दल को इस शोध अध्ययन के दौरान ऐसी जानकारी मिलती है, जो आपके अध्ययन में भाग लेने के निर्णय को जारी रखने को प्रभावित कर सकती है या कुछ संदेह बढ़ा सकती है, तो आप को उस जानकारी के बारे में बताया जायेगा ।

संपर्क व्यक्ति

अन्य जानकारी / प्रश्नों के लिए, आप निम्न पते पर संपर्क कर सकते हैं:

प्रधान अन्वेषक:

डॉ संदीप सिंह सिद्धू / मोबाइल: 9814025085
गैस्ट्रोएंटरोलॉजी विभाग
डीएमसी और अस्पताल, लुधियाना फैक्स: 0161-2302620
ईमेल: sandeepsidhu1963@gmail.com

सह-अन्वेषक

डॉ ओमेश गोयल / मोबाइल: 9914821155
गैस्ट्रोएंटरोलॉजी विभाग
डीएमसी और अस्पताल, लुधियाना फैक्स: 0161-2302620
ईमेल: goyalomesh@yahoo.co.in

विरोध के मामले में, आप हमारे संस्थागत नैतिकता समिति के **संयोजक**से निम्न पते पर संपर्क कर सकते हैं:

डॉ गगनदीप सिंह संयोजक, संस्थागत आचार समिति
डीएमसी और अस्पताल

<u>रोगी सहमति फार्म</u>

अध्ययन का शीर्षक: पूर्ण शीर्षक: बोवेन कोलोस्ट्रम बनाम प्लेसबो की तुलना: गंभीर शराब संबंधी हेपेटाइटिस के उपचार में डबल अज्ञात प्लेसबो (प्रयोगिक औषधि) का क्रमरहित नियंत्रित परीक्षण।

प्रतिभागी का नाम: ____

प्रधान / सह अन्वेषक का नाम: ___________________________

संस्था का नाम: डीएमसी और अस्पताल, लुधियाना

सूचित सहमति का दस्तावेज़ीकरण

मैंने, ..., इस प्रपत्र में लिखी जानकारी को पढ़ लिया है (या यह मेरे लिए पढ़ा गया है) । मैं किसी भी सवाल पूछने के लिए स्वतंत्र था और उसका जवाब मुझे दे दिया गया है । मैं 18 वर्षसे अधिक आयु का हूँ और अपने चुनाव करने की स्वतंत्रता का पालन करते हुए, मैंने इस अध्ययन में एक भागीदार के रूप में सम्मिलित होने के लिए अपनी सहमतिदी है, जिसका शीर्षक है: पूर्ण शीर्षक: बोवेन कोलोस्ट्रम बनाम प्लेसबो की तुलना: गंभीर शराब संबंधी हेपेटाइटिस के उपचार में डबल अज्ञात प्लेसबो (प्रयोगिक औषधि) का क्रमरहित नियंत्रित परीक्षण।

(1) मैंने इस सहमति पत्र को और उपलब्ध कराई गई जानकारी को पढ़ और समझ लिया है ।
(2) मुझे सहमति दस्तावेज़ समझाया गया है ।
(3) मुझे अध्ययन की प्रकृति के बारे में विस्तार से बताया गया है ।
(4) अपने अधिकारों और जिम्मेदारियों के बारे में अन्वेषक द्वारा मुझे समझाया गया है ।
(5) मुझे अध्ययन में मेरी भागीदारी के साथ जुड़े जोखिम के बारे में सलाह दी गई है ।
(6) मैंने पिछले 6हफ्तों में लिए गए सभी उपचारों के विषय में अन्वेषक को सूचित किया है, जिसमें देसी (वैकल्पिक) उपचार भी शामिल है ।
(7) मैं अन्वेषक के साथ सहयोग करने के लिए सहमत हूँ और असामान्य लक्षण दिखने पर मैं तुरंत उसे सूचित करूंगा ।
(8) मैंने पिछले 6 महीनों में किसी भी शोध अध्ययन में भाग नहीं लिया है ।
(9) मैं इस तथ्य से परिचित हूँ कि मैं किसी भी समय बिना कारण दिए इस अध्ययन को छोड़ सकता हूँ और यह इस अस्पताल में भविष्य में मेरे उपचार को प्रभावित नहीं करेगा ।
(10) मैं यह भी जानता हूँ कि अन्वेषक इस अध्ययन से मेरी भागीदारी किसी भी समय किसी भी कारण से बिना, मेरी सहमति के बिना समाप्त कर सकता है ।
(11) मैं जांचकर्ताओं को अनुमति देता हूँ कि इस अध्ययन में भागीदारी के परिणामस्वरुप मुझ से प्राप्त जानकारी वे नियामक अधिकारियों, सरकारी एजेंसियों, और आचार समिति कोजारी कर सकते हैं । मैं समझता हूँ कि वे मेरे मूल अभिलेखों का निरीक्षण कर सकते हैं ।
(13) मेरी पहचान गोपनीय रखी जाएगी, यदि मेरा डेटा सार्वजनिक रूप से प्रस्तुत किया जाता है ।

(14) यदि, निर्देशों का पालन करने के बावजूद, मुझे अध्ययन की योजना में निर्धारित रूप में किसी भी पदार्थ या किसी प्रक्रिया की वजह से शारीरिक रूप से नुकसान पहुंचता है, तो मेरा उपचार अनुसंधानात्मक स्थल पर नि: शुल्क किया जाएगा / प्रायोजकसभी खर्च वहन करेगा, जो बीमा एजेंसी या किसी सरकारी कार्यक्रम या किसी तीसरे पक्ष द्वारा वहन नहीं किया जायेगा ।
(15) मुझे अपने सभी प्रश्नों का उत्तर संतोषजनक रूप से मिल गया है ।
(16) मैंने इस शोध अध्ययन में रहने का निर्णय लिया है ।

मैं जानता हूं कि यदि इस अध्ययन के दौरान मुझे कोई प्रश्न पूछना है, तो मुझे ऊपर सूचीबद्ध पतों में से किसी एक पर संपर्क करना चाहिए । इस सहमति पर हस्ताक्षर करके, मैं यह प्रमाणित करता हूँ कि इस दस्तावेज़ में दी गई जानकारी मुझे स्पष्ट रूप से समझायी गयी है और मेरे द्वारा समझी गयी है । मुझे भी इस सहमति दस्तावेज की एक प्रति दी जाएगी ।

प्रतिभागी का नाम और हस्ताक्षर / अंगूठे का निशान
(या कानूनी प्रतिनिधि यदि भागीदार अक्षम है) :
________________ (नाम) ____________ (हस्ताक्षर)

दिनांक: __________ समय: ________

नाम और निष्पक्ष गवाह का हस्ताक्षर (अनपढ़ रोगियों के लिए आवश्यक) :

________________ (नाम) ____________ (हस्ताक्षर)

दिनांक: __________ समय: ________

निष्पक्ष गवाह का पता और संपर्क नंबर: ____________________
____

अन्वेषक का नाम और हस्ताक्षर या उसका सहमति प्राप्त प्रतिनिधि:

___________________ (नाम) ________________ (हस्ताक्षर)

___________________ (तिथि)

अन्वेषक का सर्टिफिकेट

मैं यह प्रमाणित करता हूँ कि इस सहमति दस्तावेज़ में वर्णित अध्ययन की प्रकृति, उद्देश्य और संभावित खतरों सहित सभी तत्वों को व्यक्ति को विस्तार से बताया गया है । मेरे मत में भागीदार / कानूनी प्रतिनिधि में इस शोध में भाग लेने के लिए सूचित सहमति देने की कानूनी क्षमता है और वह स्वेच्छा से और जानबूझकर भाग लेने के लिए सूचित सहमति देरहा है ।

अन्वेषक के हस्ताक्षर: ________________ दिनांक: __________

अन्वेषक का नाम:______________________
