# Supplementary material for: A multicenter double-blind, placebo-controlled randomized trial to evaluate the safety and efficacy of bovine colostrum in the treatment of severe alcoholic hepatitis (SAH)
Source: Trials. 2023 Aug 11;24:515. doi: 10.1186/s13063-023-07505-8 (PMC10416362; doi:10.1186/s13063-023-07505-8)
Supplement: Supplementary file 1 — Additional file 1: Annexure 1. Technological process flow chart of production of Bovine Colostrum. Annexure 2. Visit. Schedule and assessments. Annexure 3. Patient information sheet (English Version). Annexure 4. Patient consent form (in English). Annexure 5. List of study sites participating in this study. Annexure 6. Patient information sheet (Hindi & Punjabi Version). Patient consent form (Hindi & Punjabi Version). [file 13063_2023_7505_MOESM1_ESM.zip › Consent Form Punjabi (3)R2.pdf]

ਰੋਗੀ ਜਾਣਕਾਰੀ ਸ਼ੀਟ

ਤਾਰੀਖ਼:

ਖੋਜਕਰਤਾ :______________________________

(ਮੁਖ **ਅਤੇ-ਖੋਜਕਰਤਾਸਹਿ)** :______________________________

ਪ੍ਰਤੀਭਾਗੀ ਦਾ ਨਾਮ : ___________________________

ਪੂਰਾ ਹਸਰਲੇਖ: ਬੋਵਾਈਨ ਕਾਲੋਸਟ੍ਰਾਮਜ਼ ਬਨਾਮ ਪਲੇਸਬੋ ਦੀ ਤੁਲਨਾ: ਸਧਾਰਣ ਅਲਕੋਹਲ ਹੈਪੇਟਾਈਟਸ ਦੇ ਇਲਾਜ ਵਿੱਚ ਸਟ੍ਰੈਂਡਿਡ ਡਬਲ ਬਲਾਈਡ ਟਰਾਇਲ.

ਤੁਹਾਨੂੰ ਇਸ ਖੋਜ ਅਧਿਐਨ ਵਿਚ ਹਿੱਸਾ ਲੈਣ ਲਈ ਸੱਦਾ ਦਿੱਤਾ ਜਾਂਦਾ ਹੈ. ਇਸ ਦਸਤਾਵੇਜ ਵਿਚ ਦਿੱਤੀ ਜਾਣ ਵਾਲੀ ਜਾਣਕਾਰੀ ਦਾ ਮਤਲਬ ਇਹ ਹੈ ਕਿ ਤੁਸੀਂ ਇਹ ਫ਼ੈਸਲਾ ਕਰਨ ਵਿਚ ਸਹਾਇਤਾ ਕਰ ਸਕਦੇ ਹੋ ਕਿ ਤੁਸੀਂ ਹਿੱਸਾ ਲੈਣਾ ਹੈ ਜਾਂ ਨਹੀਂ. ਕਿਰਪਾ ਕਰਕੇ ਇਹ ਪੁੱਛਣ ਵਿੱਚ ਬੇਝਿਜਕ ਗੱਲ ਕਰੋ ਕਿ ਕੀ ਤੁਹਾਡੇ ਕੋਲ ਕੋਈ ਪੁੱਛਗਿੱਛ ਜਾਂ ਚਿੰਤਾਵਾਂ ਹਨ?

ਤੁਹਾਨੂੰ ਡੀ ਐਮ ਸੀ ਅਤੇ ਹਸਪਤਾਲ, ਲੁਧਿਆਣਾ, ਪੰਜਾਬ ਵਿੱਚ ਆਯੋਜਿਤ ਕੀਤੇ ਜਾ ਰਹੇ ਇਸ ਅਧਿਐਨ ਵਿੱਚ ਭਾਗ ਲੈਣ ਲਈ ਕਿਹਾ ਜਾ ਰਿਹਾ ਹੈ ਕਿਉਂਕਿ ਤੁਸੀਂ ਆਪਣੀ ਯੋਗਤਾ ਦੇ ਮਾਪਦੰਡ ਨੂੰ ਪੂਰਾ ਕਰਦੇ ਹੋ: 18 ਸਾਲ ਤੋਂ ਘੱਟ ਉਮਰ ਦੇ ਗੰਭੀਰ ਮੈਡੀਕੋਡ ਹੈਪੇਟਾਈਟਸ ਦਾ ਨਿਦਾਨ, ਅਲਿਐਨ ਵਿੱਚ ਵਰਤੇ ਗਏ ਏਜੰਟਾਂ ਦੀ ਵਰਤੋਂ ਪ੍ਰਤੀ ਕੋਈ ਵਰੋਤਮਕਤਾ ਨਹੀਂ ਪਾਈ ਗਈ, ਲਜਸਦਾ ਅਰਥ ਬੋਵਾਇਨ ਕੋਲੋਸਟਰਮ, ਪ੍ਰਤੀ ਲਗਆਤ ਅਲਰਜੀ ਦੀ ਗੈਰ-ਮੌਜੂਦਗੀ ਹੈ।

ਤੁਸੀਂ ਇਸ ਅਧਿਐਨ ਵਿਚ ਭਰਤੀ ਕਰਨ ਵਾਲੇ 250 ਮਰੀਜ਼ਾਂ ਵਿਚੋਂ ਇਕ ਹੋਵੋਗੇ. ਤੁਹਾਨੂੰ 4 ਹਫਤਿਆਂ ਲਈ ਫਰੀਜ਼ ਸੁਕਾਏ ਪਾਊਡਰ ਜਾਂ ਪਲੇਸਬੋ (20 ਗ੍ਰਾਮ ਤਿੰਨ ਵਾਰ ਇੱਕ ਦਿਨ) ਦੇ ਰੂਪ ਵਿੱਚ ਮੌਸਿਕ ਪਾਚੁਰਾਈਜ਼ਡ ਬੋਵਾਈਨ ਕੌਲੋਸਟਰਮ ਦਿੱਤਾ ਜਾਵੇਗਾ

ਖੋਜ ਦਾ ਮਕਸਦ ਕੀ ਹੈ?

ਗਰੀਕ੍ਰੋਕੋਰਟੀਕਾਈਡਜ਼ ਅਤੇ ਪੈਂਟੋਸੀਪੀਹਿਲਾਲਾਈਨ ਵਰਗੇ ਮੌਜੂਦਾ ਇਲਾਜ ਜਿਵੇਂ ਜੀਵਾਣੂਆਂ ਨਾਲ ਪੀੜਤ ਰਹਿਣ ਵਿਚ ਪਹਿਲੇ ਮਹੀਨਿਆਂ ਵਿਚ ਬਹੁਤ ਜ਼ਿਆਦਾ ਮਾਤਰਾ ਵਿਚ ਹੋਣ ਵਾਲੀ ਹੈਪੇਟਾਈਟਸ ਦੀ ਦਰ ਬਹੁਤ ਉੱਚੀ ਦਰ (30-40%) ਨਾਲ ਜੁੜੀ ਹੋਈ ਹੈ. ਇੱਕ ਦੀ ਮੌਤ ਨੂੰ ਰੋਕਣ ਲਈ ਇਲਾਜ ਕਰਨ ਲਈ ਲੋੜੀਂਦੇ ਮਰੀਜ਼ਾਂ ਦੀ ਗਿਣਤੀ 5 ਹੈ. ਇਸ ਲਈ ਸਾਨੂੰ ਸਾਕਾਰ ਪ੍ਰਭਾਵਾਂ ਨੂੰ ਵਧਾਏ ਬਿਨਾਂ ਬਚਾਅ ਦੀ ਦਰ ਨੂੰ ਬਿਹਤਰ ਬਣਾਉਣ ਲਈ ਇੱਕ ਬਿਹਤਰ ਇਲਾਜ ਦੀ ਲੋੜ ਹੈ

ਅਹਿਐਨ ਦਾ ਹਿਜ਼ਾਇਨ

ਅਧਿਐਨ ਵਿਚਲੇ ਸਾਰੇ ਰੋਗੀਆਂ ਨੂੰ 4 ਹਫਤਿਆਂ ਲਈ ਫਰੀਜ਼ ਸੁਕਾਏ ਪਾਊਡਰ ਜਾਂ ਪਲੇਸਬੋ (20 ਗ੍ਰਾਮ ਤਿੰਨ ਵਾਰ ਇਕ ਦਿਨ) ਦੇ ਤੌਰ ਤੇ ਮੌਜ਼ਲ ਪਾਚੁਰਾਈਜ਼ਡ ਬੋਵਾਈਨ ਕੋਲੋਸਟ੍ਰਮ ਦਿੱਤਾ ਜਾਵੇਗਾ

ਅਹਿਐਨ ਪਰਹਕਹਰਆਵਾਂ

ਅਧਿਐਨ ਵਿੱਚ ਜੀਵਣ ਦੀ ਦਰ ਅਤੇ ਐਮ ਡੀ ਐੱਫ ਸਕੋਰ ਨੂੰ ਸੁਧਾਰਨ ਵਿੱਚ ਮੌਖਿਕ ਬੋਵਾਈਨ ਕੋਲੋਸਟਮ ਦੇ ਪ੍ਰਭਾਵ ਦਾ ਮੁਲਾਂਕਣ ਕਰਨਾ ਸ਼ਾਮਲ ਹੈ. ਇੱਕ ਵਾਰ ਜਦੋਂ ਮਰੀਜ਼ ਨੂੰ ਅਧਿਐਨ ਵਿੱਚ ਦਾਖਲ ਕੀਤਾ ਜਾਂਦਾ ਹੈ, ਤਾਂ ਉਸ ਨੂੰ ਹਦਾਇਤਾਂ ਦੀ ਪਾਲਣਾ ਕਰਨ ਦੀ ਜ਼ਰੂਰਤ ਹੁੰਦੀ ਹੈ ਜਿਵੇਂ ਕਿ ਅਧਿਐਨ ਦੀਆਂ ਦਵਾਈਆਂ ਜਿਵੇਂ ਕਿ ਹਿਦਾਇਤ ਦਿੱਤੀ ਗਈ ਹੈ, ਜਿਵੇਂ ਕਿ ਨਿਰਦੇਸ਼ ਦਿੱਤੇ ਅਨੁਸਾਰ ਖੁਰਾਕ ਲੈਣਾ. ਐਲਐਫਟੀ ਲਈ ਨਮੂਨੇ ਬੇਸਲਾਈਨ ਅਤੇ 7 ਵੇਂ ਦਿਨ, 14 ਦਿਨ, 21 ਦਿਨ, 28 ਦਿਨ ਅਤੇ ਫਿਰ ਦੋ ਮਹੀਨੇ ਜਾਂ ਇਸ ਤੋਂ ਪਹਿਲਾਂ ਦੇ ਲਈ ਨਮੂਨੇ ਲਏ ਜਾਣਗੇ ਜੇਕਰ ਸੰਕੇਤ ਮਿਲੇ ਹਨ. ਦੂਜੀ ਰੁਟੀਨ ਦੀ ਜਾਂਚ ਉਦੋਂ ਕੀਤੀ ਜਾਵੇਗੀ ਜਦੋਂ ਸੰਕੇਤ ਮਿਲੇਗਾ

ਪਰਸਵ ਦੀਸੰ ਭਾਵਨਾ ਵਾਲੀਆਂ ਔਰਤਾਂ

ਔਰਤਾਂ ਜੇ ਉਹ ਗਰਭਵਤੀ ਹੈ ਜਾਂ ਬੱਚੇ ਨੂੰ ਛਾਤੀ ਦਾ ਦੁੱਧ ਚੁੰਘਾਉਂਦੀ ਹੈ ਤਾਂ ਇੱਕ ਮਰੀਜ਼ ਨੂੰ ਭਾਗ ਨਹੀਂ ਲੈਣਾ ਚਾਹੀਦਾ.

ਤੁਹਾਡੇ ਲਈ ਸੰਭਾਵੀ ਜੋਖਮ

ਅਧਿਐਨ ਤੋਂ ਪਹਿਲਾਂ ਦੇ ਅਧਿਐਨਾਂ ਵਿਚ, ਡਰੱਗ ਐਲਰਜੀ ਅਤੇ ਲੈਂਕੌਂਸ ਅਸਹਿਣਸ਼ੀਲਤਾ ਵਾਲੇ ਮਰੀਜ਼ਾਂ ਦੇ ਇਲਾਵਾ ਮਾੜੇ ਪ੍ਰਭਾਵਾਂ ਦਾ ਪ੍ਰਦਰਸ਼ਨ ਨਹੀਂ ਕੀਤਾ ਗਿਆ.

ਤੁਹਾਡੇ ਲਈ ਸੰਭਵ ਲਾਭ

ਇਲਾਜ ਖੋਜ ਦੇ ਲਾਭ ਤੋਂ ਇਲਾਵਾ, ਤੁਹਾਨੂੰ ਇਸ ਖੋਜ ਅਧਿਐਨ ਤੇ ਹੋਣ ਦਾ ਕੋਈ ਫਾਇਦਾ ਲੈਣ ਦੀ ਉਮੀਦ ਨਹੀਂ ਕੀਤੀ ਜਾਂਦੀ.

ਹੋਰ ਲੋਕਾਂ ਨੂੰ ਸੰਭਵ ਲਾਭ

ਖੋਜ ਦੇ ਨਤੀਜੇ ਵਿਕਾਸ ਦੇ ਰੂਪ ਵਿਚ ਸਮਾਜ ਨੂੰ ਲਾਭ ਪ੍ਰਦਾਨ ਕਰ ਸਕਦੇ ਹਨ
ਭਵਿੱਖ ਦੇ ਮਰੀਜ਼ਾਂ ਨੂੰ ਮੈਡੀਕਲ ਗਿਆਨ ਅਤੇ ਇਲਾਜ ਦੇ ਲਾਭ

ਤੁਹਾਡੇ ਕੋਲ ਵਿਕਲਪ ਹਨ

ਜੇ ਤੁਸੀਂ ਹਿੱਸਾ ਨਹੀਂ ਲੈਣਾ ਚਾਹੁੰਦੇ ਹੋ, ਤਾਂ ਤੁਹਾਡੇ ਕੋਲ ਤੁਹਾਡੀ ਬਿਮਾਰੀ ਦੇ ਮਿਆਰੀ ਇਲਾਜ ਦੀ ਚੋਣ ਕਰਨ ਦਾ ਵਿਕਲਪ ਹੁੰਦਾ ਹੈ.

ਭਾਗੀਦਾਰ ਨੂੰ ਖਰਚਾ

ਤੁਹਾਨੂੰ ਇਸ ਖੋਜ ਅਧਿਐਨ ਵਿਚ ਹਿੱਸਾ ਲੈਣ ਲਈ ਭੁਗਤਾਨ ਨਹੀਂ ਕੀਤਾ ਜਾਵੇਗਾ. ਅਧਿਐਨ ਦਵਾਈਆਂ ਦੇ ਕਾਰਨ ਆਉਣ ਵਾਲੀ ਕਿਸੇ ਵੀ ਗਲਤ ਘਟਨਾ ਦੇ ਮਾਮਲੇ ਵਿੱਚ, ਤੁਹਾਨੂੰ ਸਾਡੇ ਇੰਸਟੀਚਿਊਟ ਵਿੱਚ ਮੁਫਤ ਇਲਾਜ ਅਤੇ ਜੇ ਲੋੜ ਹੋਵੇ ਤਾਂ ਸਹੀ ਰੈਫਰਲ ਮੁਹੱਈਆ ਕਰਵਾਇਆ ਜਾਵੇਗਾ.
ਇਸ ਖੋਜ ਅਧਿਐਨ ਦੌਰਾਨ ਸੱਟ-ਫੇਟ ਜਾਂ ਕਿਸੇ ਡਾਕਟਰੀ ਸਮੱਸਿਆ ਦੇ ਮਾਮਲੇ ਵਿਚ ਤੁਹਾਨੂੰ ਕੀ ਕਰਨਾ ਚਾਹੀਦਾ ਹੈ?
ਤੁਹਾਡੀ ਸੁਰੱਖਿਆ ਖੋਜ ਦੀ ਮੁੱਖ ਚਿੰਤਾ ਹੈ. ਜੇ ਇਸ ਅਧਿਐਨ ਵਿਚ ਹੋਣ ਦੇ ਨਤੀਜੇ ਵਜੋਂ ਤੁਹਾਡੀ ਕੋਈ ਡਾਕਟਰੀ ਸਮੱਸਿਆ ਹੈ, ਤਾਂ ਤੁਹਾਨੂੰ ਸਹਿਮਤੀ ਫਾਰਮ ਦੇ ਅੰਤ ਵਿਚ ਸੂਚੀਬੱਧ ਲੋਕਾਂ ਵਿਚੋਂ ਕਿਸੇ ਨਾਲ ਸੰਪਰਕ ਕਰਨਾ ਚਾਹੀਦਾ ਹੈ. ਤੁਹਾਨੂੰ ਲੋੜੀਂਦੀ ਦੇਖਭਾਲ / ਇਲਾਜ ਪ੍ਰਦਾਨ ਕੀਤਾ ਜਾਵੇਗਾ. ਇਸ ਤੋਂ ਇਲਾਵਾ ਤੁਸੀਂ ਆਪਣੇ ਕਾਨੂੰਨੀ ਅਧਿਕਾਰਾਂ ਦੇ ਹੱਕਦਾਰ ਹੋਵੋਗੇ.

ਤੁਹਾਡੇ ਤੋਂ ਪ੍ਰਾਪਤ ਕੀਤੀ ਗਈ ਜਾਣਕਾਰੀ ਦੀ ਗੁਪਤਤਾ

ਤੁਹਾਡੇ ਕੋਲ ਤੁਹਾਡੀ ਮੈਡੀਕਲ ਜਾਣਕਾਰੀ ਦੀ ਪਰਾਈਵੇਸੀ (ਨਿੱਜੀ ਵੇਰਵੇ, ਸਰੀਰਕ ਪ੍ਰੀਖਿਆਵਾਂ, ਜਾਂਚਾਂ, ਅਤੇ ਤੁਹਾਡੇ ਡਾਕਟਰੀ ਇਤਿਹਾਸ ਦੇ ਨਤੀਜੇ) ਬਾਰੇ ਗੁਪਤਤਾ ਦਾ ਅਧਿਕਾਰ ਹੈ. ਇਸ ਦਸਤਾਵੇਜ਼ 'ਤੇ ਦਸਤਖਤ ਕਰਕੇ, ਜੇਕਰ ਲੋੜ ਪਵੇ ਤਾਂ ਤੁਹਾਡੇ ਡੇਟਾ ਨੂੰ ਵੇਖਣ ਲਈ ਤੁਸੀਂ ਖੋਜ ਟੀਮ ਦੇ ਪੜਤਾਲਕਰਤਾਵਾਂ, ਹੋਰ ਸਟੱਡੀ ਕਰਮਚਾਰੀਆਂ, ਸੰਸਥਾਗਤ ਨੈਿਤਕ ਕਮੇਟੀ ਅਤੇ ਭਾਰਤ ਦੇ ਨਸ਼ੀਲੇ ਪਦਾਰਥ ਕੰਟਰੋਲਰ ਜਨਰਲ ਦੀ ਤਰਾਂ ਕਾਨੂੰਨ ਦੁਆਰਾ ਲੋੜੀਂਦੇ ਕਿਸੇ ਵਿਅਕਤੀ ਜਾਂ ਏਜੰਸੀ ਨੂੰ ਇਜਾਜ਼ਤ ਦੇ ਰਹੇ ਹੋਵੋਗੇ.
ਇਸ ਖੋਜ ਦੇ ਹਿੱਸੇ ਵਜੋਂ ਕੀਤਾ ਗਿਆ ਕਲੀਨਿਕਲ ਟੈਸਟ ਅਤੇ ਥੈਰੇਪੀ ਦੇ ਨਤੀਜੇ ਤੁਹਾਡੇ ਮੈਡੀਕਲ ਰਿਕਾਰਡ ਵਿੱਚ ਸ਼ਾਮਿਲ ਕੀਤੇ ਜਾ ਸਕਦੇ ਹਨ. ਇਸ ਅਧਿਐਨ ਦੀ ਜਾਣਕਾਰੀ, ਜੇਕਰ ਵਿਗਿਆਨਕ ਰਸਾਲਿਆਂ ਵਿੱਚ ਪ੍ਰਕਾਸ਼ਿਤ ਕੀਤੀ ਗਈ ਹੈ ਜਾਂ ਵਿਗਿਆਨਕ ਮੀਟਿੰਗਾਂ ਵਿੱਚ ਪੇਸ਼ ਕੀਤੀ ਗਈ ਹੈ, ਤਾਂ ਤੁਹਾਡੀ ਪਛਾਣ ਪ੍ਰਗਟ ਨਹੀਂ ਕੀਤੀ ਜਾਏਗੀ.

ਅਧਿਐਨ ਵਿਚ ਹਿੱਸਾ ਨਾ ਲੈਣ ਦੇ ਤੁਹਾਡੇ ਫ਼ੈਸਲੇ 'ਤੇ ਤੁਹਾਨੂੰ ਕੀ ਅਸਰ ਪਏਗਾ?

ਇਸ ਖੋਜ ਅਧਿਐਨ ਵਿੱਚ ਹਿੱਸਾ ਨਾ ਲੈਣ ਦਾ ਤੁਹਾਡਾ ਫੈਸਲਾ ਤੁਹਾਡੀ ਡਾਕਟਰੀ ਦੇਖਭਾਲ ਜਾਂ ਖੋਜਕਰਤਾ ਜਾਂ ਸੰਸਥਾ ਨਾਲ ਤੁਹਾਡੇ ਸਬੰਧ ਨੂੰ ਪ੍ਰਭਾਵਤ ਨਹੀਂ ਕਰੇਗਾ. ਤੁਹਾਡਾ ਡਾਕਟਰ ਅਜੇ ਵੀ ਤੁਹਾਡੀ ਦੇਖਭਾਲ ਕਰੇਗਾ ਅਤੇ ਤੁਸੀਂ ਉਨ੍ਹਾਂ ਲਾਭਾਂ ਨੂੰ ਨਹੀਂ ਗੁਆਓਗੇ ਜਿਨ੍ਹਾਂ ਦੇ ਤੁਸੀਂ ਹੱਕਦਾਰ ਹੋ

ਕੀ ਤੁਸੀਂ ਇਹ ਫੈਸਲਾ ਕਰ ਸਕਦੇ ਹੋ ਕਿ ਸਟੱਡੀ ਸ਼ੁਰੂ ਕਰਨ ਤੋਂ ਬਾਅਦ ਤੁਸੀਂ ਹਿੱਸਾ ਲੈਣਾ ਬੰਦ ਕਰ ਸਕਦੇ ਹੋ?
ਇਸ ਖੋਜ ਵਿਚ ਹਿੱਸਾ ਲੈਣ ਦਾ ਭਾਵ ਪੂਰੀ ਤਰਾਂ ਸਵੈਇੱਛਤ ਹੈ ਅਤੇ ਤੁਹਾਨੂੰ ਕਿਸੇ ਵੀ ਕਾਰਨ ਦਿੱਤੇ ਬਗੈਰ ਕਿਸੇ ਵੀ ਸਮੇਂ ਅਧਿਐਨ ਦੇ ਦੌਰਾਨ ਕਿਸੇ ਵੀ ਸਮੇਂ ਇਸ ਅਧਿਐਨ ਤੋਂ ਵਾਪਸ ਲੈਣ ਦਾ ਹੱਕ ਹੈ. ਪਰ, ਇਹ ਸਲਾਹ ਦਿੱਤੀ ਜਾਂਦੀ ਹੈ ਕਿ ਤੁਸੀਂ ਇਲਾਜ ਰੋਕਣ ਤੋਂ ਪਹਿਲਾਂ ਰਿਸਰਚ ਟੀਮ ਨਾਲ ਗੱਲ ਕਰੋ. ਹਾਲਾਂਕਿ ਇਹ ਸਲਾਹ ਦਿੱਤੀ ਜਾਂਦੀ ਹੈ ਕਿ ਤੁਸੀਂ ਜਾਂਚਕਾਰਾਂ ਨੂੰ ਵਾਪਸ ਲੈਣ ਦੇ ਕਾਰਨ ਦੇ ਦਿਓ, ਇਹ ਲਾਜ਼ਮੀ ਨਹੀਂ ਹੈ.

ਕੀ ਜਾਂਚਕਾਰ ਤੁਹਾਨੂੰ ਅਧਿਐਨ ਤੋਂ ਹਟਾ ਸਕਦਾ ਹੈ?
ਜੇ ਤੁਸੀਂ ਜਾਂਚਕਾਰਾਂ ਜਾਂ ਖੋਜ ਟੀਮ ਦੀਆਂ ਹਦਾਇਤਾਂ ਦੀ ਪਾਲਣਾ ਨਹੀਂ ਕਰਦੇ ਜਾਂ ਜੇ ਖੋਜਕਰਤਾ ਸੋਚਦਾ ਹੈ ਕਿ ਅੱਗੇ ਦੀ ਹਿੱਸੇਦਾਰੀ ਤੁਹਾਨੂੰ ਨੁਕਸਾਨ ਪਹੁੰਚਾ ਸਕਦੀ ਹੈ ਤਾਂ ਤੁਹਾਨੂੰ ਤੁਹਾਡੀ ਸਹਿਮਤੀ ਦੇ ਬਗੈਰ ਅਧਿਐਨ ਬੰਦ ਕੀਤਾ ਜਾ ਸਕਦਾ ਹੈ.

ਨਵੀਂ ਜਾਣਕਾਰੀ ਦਾ ਅਧਿਕਾਰ
ਜੇ ਖੋਜ ਟੀਮ ਨੂੰ ਇਸ ਖੋਜ ਅਧਿਐਨ ਦੌਰਾਨ ਕੋਈ ਨਵੀਂ ਜਾਣਕਾਰੀ ਪ੍ਰਾਪਤ ਹੋ ਸਕਦੀ ਹੈ ਜੋ ਅਧਿਐਨ ਵਿਚ ਹਿੱਸਾ ਲੈਣ ਲਈ ਤੁਹਾਡੇ ਫ਼ੈਸਲੇ 'ਤੇ ਅਸਰ ਪਾ ਸਕਦੀ ਹੈ, ਜਾਂ ਕੁਝ ਸ਼ੰਕਿਆਂ ਨੂੰ ਉਠਾ ਸਕਦੀ ਹੈ, ਤਾਂ ਤੁਹਾਨੂੰ ਇਸ ਜਾਣਕਾਰੀ ਬਾਰੇ ਦੱਸਿਆ ਜਾਵੇਗਾ.

ਸੰਪਰਕ ਵਿਅਕਤੀ

ਵਧੇਰੇ ਜਾਣਕਾਰੀ ਲਈ / ਸਵਾਲਾਂ ਲਈ, ਤੁਸੀਂ ਸਾਡੇ ਨਾਲ ਹੇਠ ਲਿਖੇ ਪਤੇ ਤੇ ਸੰਪਰਕ ਕਰ ਸਕਦੇ ਹੋ:

ਪ੍ਰਿੰਸੀਪਲ ਇਨਵੈਸਟੀਗੇਟਰ: ਡਾ. ਸੰਦੀਪ ਸਿੰਘ ਸਿੱਧੂ / ਫੋਨ: 9814025085

ਗੈਸਟ੍ਰੋਏਟਰੋਲੋਜੀ ਵਿਭਾਗ ਡੀ ਐਮ ਸੀ ਅਤੇ ਹਸਪਤਾਲ, ਲੁਧਿਆਣਾ

ਫੈਕਸ: 0161-2302620

ਈਮੇਲ: ਸੰਦੀਪਸਿਡਹੁ1963@gmail.com

ਕੋ-ਇਨਵੈਸਟੀਗੇਟਰ ਡਾ ਓਮੇਸ਼ ਗੋਇਲ / ਫੋਨ: 9914821155

ਗੈਸਟ੍ਰੋਏਟਰੋਲੋਜੀ ਵਿਭਾਗ ਡੀ ਐਮ ਸੀ ਅਤੇ ਹਸਪਤਾਲ, ਲੁਧਿਆਣਾ

ਫੈਕਸ: 0161-2302620

ਈਮੇਲ: goyalomeah@yahoo.co.in

ਅਪਵਾਦਾਂ ਦੇ ਮਾਮਲੇ ਵਿਚ, ਤੁਸੀਂ ਸਾਡੀ ਸੰਸਥਾਗਤ ਨੈਤਿਕਤਾ ਦੇ ਕਨਵੀਨਰ ਨਾਲ ਸੰਪਰਕ ਕਰ ਸਕਦੇ ਹੋ ਕਮੇਟੀ ਨੂੰ ਹੇਠ ਲਿਖੇ ਪਤੇ 'ਤੇ:

ਡਾ ਗਗਨਦੀਪ ਸਿੰਘ ਕਨਵੀਨਰ,

ਸੰਸਥਾਗਤ ਨੈਤਿਕਤਾ ਕਮੇਟੀ

ਡੀ ਐਮ ਸੀ ਅਤੇ ਹਸਪਤਾਲ

ਟੈਲੀਫੋਨ: 9815500720

ਰੋਗੀ ਸਹਿਮਤੀ ਫਾਰਮ

ਅਧਿਐਨ ਦਾ ਸਿਰਲੇਖ: ਬੋਵਾਈਨ ਕਾਲੋਸਟ੍ਰਾਮਜ਼ ਬਨਾਮ ਪਲੇਸਬੋ ਦੀ ਤੁਲਨਾ: ਸਧਾਰਣ ਅਲਕੋਹਲ ਹੈਪੇਟਾਈਟਸ ਦੇ ਇਲਾਜ ਵਿੱਚ ਸਟ੍ਰੈਂਡਿਡ ਡਬਲ ਬਲਾਈਡ ਟਰਾਇਲ.

ਪ੍ਰਤੀਭਾਗੀ ਦਾ ਨਾਮ: ____

ਮੁੱਖ/ਸਹਿ-ਖੋਜਕਰਤਾ ਦਾ ਨਾਮ: ______________________________

ਸੰਸਥਾ ਦਾ ਨਾਮ: ਡੀਐਮਸੀ ਅਤੇ ਹਸਪਤਾਲ, ਲੁਧਿਆਣਾ

ਸੂਚਿਤ ਸਹਿਮਤੀ ਦਾ ਦਸਤਾਵੇਜ਼ੀਕਰਨ

ਮੈਂ, … … … … … … … … … ., ਇਸ ਫਾਰਮ ਵਿਚਲੀ ਜਾਣਕਾਰੀ ਪੜ੍ਹ ਲਈ ਹੈ (ਜਾਂ ਇਹ ਮੈਨੂੰ ਪੜ੍ਹ ਕੇ ਦੱਸੀ ਗਈ ਹੈ)। ਮੈਂ ਕੋਈ ਵੀ ਪ੍ਰਸ਼ਨ ਪੁੱਛਣ ਲਈ ਸੁਤੰਤਰ ਸੀ ਅਤੇ ਉਹਨਾਂ ਨੇ ਉੱਤਰ ਦਿੱਤੇ ਮੇਰੀ ਉਮਰ 18 ਸਾਲ ਤੋਂ ਵੱਧ ਹੈ ਅਤੇ ਮੈਂ ਆਪਣੀ ਚੋਣ ਦੀ ਮੁਕਤ ਪਾਵਰ ਦੀ ਵਰਤੋਂ ਕਰਦਾ/ਕਰਦੀ ਹਾਂ, ਇਸ ਦੁਆਰਾ ਮੈਂ ਅਧਿਐਨ ਸਿਰਲੇਖ: 'ਬੋਵਾਈਨ ਕਾਲੋਸਟ੍ਰਾਮਜ਼ ਬਨਾਮ ਪਲੇਸਬੋ ਦੀ ਤੁਲਨਾ: ਸਧਾਰਣ ਅਲਕੋਹਲ ਹੈਪੇਟਾਈਟਸ ਦੇ ਇਲਾਜ ਵਿੱਚ ਸਟ੍ਰੈਂਡਿਡ ਡਬਲ ਬਲਾਈਡ ਟਰਾਇਲ ਪਰੀਖਣ ਵਿੱਚ ਪ੍ਰਤੀਭਾਗੀ ਦੇ ਤੌਰ ਤੇ ਸ਼ਾਮਿਲ ਕਰਨ ਲਈ ਸਹਿਮਤੀ ਦਿੰਦਾ/ਦਿੰਦੀ ਹਾਂ।

(1) ਮੈਂ ਸਹਿਮਤੀ ਫਾਰਮ ਅਤੇ ਦਿੱਤੀ ਗਈ ਜਾਣਕਾਰੀ ਨੂੰ ਪੜ੍ਹ ਅਤੇ ਸਮਝ ਲਿਆ ਹੈ।

(2) ਮੈਨੂੰ ਸਹਿਮਤੀ ਦਸਤਾਵੇਜ਼ ਦਾ ਵਰਣਨ ਕੀਤਾ ਗਿਆ।

(3) ਮੈਨੂੰ ਅਧਿਐਨ ਦੀ ਪ੍ਰਕਿਰਤੀ ਬਾਰੇ ਦੱਸਿਆ ਗਿਆ।

(4) ਖੋਜਕਰਤਾ ਨੇ ਮੇਰੇ ਅਧਿਕਾਰ ਅਤੇ ਜ਼ਿੰਮੇਵਾਰੀਆਂ ਬਾਰੇ ਦੱਸਿਆ।

(5) ਮੈਨੂੰ ਅਧਿਐਨ ਵਿੱਚ ਮੇਰੀ ਭਾਗੀਦਾਰੀ ਨਾਲ ਜੁੜੇ ਖ਼ਤਰਿਆਂ ਬਾਰੇ ਦੱਸਿਆ ਗਿਆ।

(6) ਮੈਂ ਖੋਜਕਰਤਾ ਨੂੰ ਉਹਨਾਂ ਕੋਈ ਹਰ *ਦੇਸੀ* (ਵਿਕਲਪਿਕ) ਇਲਾਜ ਸਮੇਤ ਇਲਾਜਾਂ ਬਾਰੇ ਦੱਸਿਆ ਜੋ ਮੈਂ ਲੈ ਰਿਹਾ ਹਾਂ ਜਾਂ ਪਿਛਲੇ *6 ਹਫ਼ਤਿਆਂ* ਵਿੱਚ ਲਏ ਹਨ।

(7) ਮੈਂ ਖੋਜਕਰਤਾ ਦਾ ਸਹਿਯੋਗ ਦੇਣ ਪ੍ਰਤੀ ਸਹਿਮਤੀ ਪ੍ਰਗਟ ਕਰਦਾ/ਕਰਦੀ ਹਾਂ ਅਤੇ ਜੇ ਮੈਂ ਅਸਧਾਰਨ ਲੱਛਣਾਂ ਨਾਲ ਪ੍ਰਭਾਵਿਤ ਹੁੰਦਾ/ਹੁੰਦੀ ਹਾਂ, ਤਾਂ ਮੈਂ ਤੁਰੰਤ ਉਹਨਾਂ ਨੂੰ ਸੂਚਿਤ ਕਰਾਂਗਾ/ਕਰਾਂਗੀ।

(8) ਮੈਂ ਪਿਛਲੇ 6 ਮਹੀਨਿਆਂ ਵਿੱਚ ਕਿਸੇ ਵੀ ਖੋਜ ਅਧਿਐਨ ਵਿੱਚ ਭਾਗ ਨਹੀਂ ਲਿਆ।

(9) ਮੈਂ ਇਸ ਤੱਥ ਤੋਂ ਜਾਣੂੰ ਹਾਂ ਕਿ ਮੈਂ ਬਿਨਾਂ ਕੋਈ ਕਾਰਨ ਦਿੱਤੇ ਕਿਸੇ ਵੀ ਸਮੇਂ ਅਧਿਐਨ ਛੱਡ ਸਕਦਾ ਹਾਂ ਅਤੇ ਇਹ ਹਸਪਤਾਲ ਵਿੱਚ ਮੇਰੇ ਭਵਿੱਖ ਦੇ ਇਲਾਜ ਨੂੰ ਪ੍ਰਭਾਵਿਤ ਨਹੀਂ ਕਰੇਗਾ।

(10) ਮੈਂ ਇਸ ਬਾਰੇ ਵੀ ਜਾਣੂੰ ਹਾਂ ਕਿ ਖੋਜਕਰਤਾ ਮੇਰੀ ਸਹਿਮਤੀ ਤੋਂ ਬਿਨਾਂ, ਕਿਸੇ ਵੀ ਕਾਰਨ ਵਜੋਂ ਅਧਿਐਨ ਵਿੱਚ ਮੇਰੀ ਭਾਗਦਾਰੀ ਸਮਾਪਤ ਕਰ ਸਕਦੇ ਹਨ।

(11) ਮੈਂ ਇਸ ਦੁਆਰਾ ਖੋਜਕਰਤਾਵਾਂ ਨੂੰ ਇਸ ਅਧਿਐਨ ਵਿੱਚ ਮੇਰੀ ਭਾਗੀਦਾਰੀ ਦੇ ਨਤੀਜੇ ਵਜੋਂ ਮੇਰੇ ਤੋਂ ਪ੍ਰਾਪਤ ਜਾਣਕਾਰੀ ਰੈਗੂਲੇਟਰੀ ਅਧਿਕਾਰੀਆਂ, ਸਰਕਾਰੀ ਏਜੰਸੀਆਂ ਅਤੇ ਨੈਤਿਕਤਾ ਕਮੇਟੀ ਨੂੰ ਦੇਣ ਦੀ ਆਗਿਆ ਦਿੰਦਾ/ਦਿੰਦੀ ਹਾਂ। ਮੈਂ ਸਮਝਦਾ/ਸਮਝਦੀ ਹਾਂ ਕਿ ਉਹ ਮੇਰੇ ਮੂਲ ਦਸਤਾਵੇਜ਼ਾਂ ਦੀ ਜਾਂਚ ਕਰ ਸਕਦੇ ਹਨ।

(13) ਜੇਕਰ ਮੇਰਾ ਡਾਟਾ ਜਨਤਕ ਤੌਰ ਤੇ ਪੇਸ਼ ਕੀਤਾ ਗਿਆ, ਤਾਂ ਮੇਰੀ ਪਹਿਚਾਣ ਗੁਪਤ ਰੱਖੀ ਜਾਵੇਗੀ।

(14) ਜੇਕਰ, ਹੇਠਾਂ ਦਿੱਤੀਆਂ ਹਿਦਾਇਤਾਂ ਦੇ ਬਾਵਜੂਦ, ਅਧਿਐਨ ਪਲਾਨ ਵਿੱਚ ਨਿਰਧਾਰਿਤ ਕਿਸੇ ਪਦਾਰਥ ਜਾਂ ਕਿਸੇ ਪ੍ਰਕਿਰਿਆ ਕਾਰਨ ਮੈਨੂੰ ਸਰੀਰਿਕ ਤੌਰ ਤੇ ਨੁਕਸਾਨ ਪਹੁੰਚਦਾ ਹੈ, ਤਾਂ ਖੋਜ ਸੰਬੰਧੀ ਸਥਾਨ ਤੇ ਮੇਰਾ ਮੁਫ਼ਤ ਇਲਾਜ ਕੀਤਾ ਜਾਵੇਗਾ। ਪ੍ਰਯੋਜਕ ਸਾਰੇ ਖ਼ਰਚੇ ਵਹਿਣ ਕਰਨਗੇ, ਜੇਕਰ ਖਰਚੇ ਮੇਰੀ ਸਰਕਾਰੀ ਏਜੰਸੀ ਜਾਂ ਸਰਕਾਰੀ ਪ੍ਰੋਗਰਾਮ ਵਹਿਣ ਨਹੀਂ ਕੀਤੇ ਜਾਂਦੇ।

(15) ਮੈਨੂੰ ਮੇਰੇ ਪ੍ਰਸ਼ਨਾਂ ਦੇ ਸੰਤੋਸ਼ਜਨਕ ਉੱਤਰ ਮਿਲੇ।

(16) ਮੈਂ ਖੋਜ ਅਧਿਐਨ ਵਿੱਚ ਭਾਗ ਲੈਣ ਦਾ ਨਿਰਣਾ ਲਿਆ ਹੈ।

ਮੈਂ ਜਾਣੂੰ ਹਾਂ ਕਿ ਜੇਕਰ ਇਸ ਅਧਿਐਨ ਦੌਰਾਨ ਮੇਰਾ ਕੋਈ ਪ੍ਰਸ਼ਨ ਹੋਵੇਗਾ ਤਾਂ ਮੈਨੂੰ ਉਪਰੋਕਤ ਪਤਿਆਂ ਵਿੱਚੋਂ ਇੱਕ ਨਾਲ ਸੰਪਰਕ ਕਰਨਾ ਪਵੇਗਾ। ਇਸ ਸਹਿਮਤੀ ਫਾਰਮ ਤੇ ਹਸਤਾਖ਼ਰ ਕਰਕੇ, ਮੈਂ ਤਸਦੀਕ ਕਰਦਾ/ਕਰਦੀ ਹਾਂ ਕਿ ਇਸ ਦਸਤਾਵੇਜ਼ ਵਿੱਚ ਦਿੱਤੀ ਜਾਣਕਾਰੀ ਦਾ ਮੈਨੂੰ ਸਪੱਸ਼ਟ ਤਰੀਕੇ ਨਾਲ ਵਰਣਨ ਕੀਤਾ ਗਿਆ ਅਤੇ ਮੈਨੂੰ ਸਪੱਸ਼ਟ ਸਮਝਾਈ ਗਈ। ਮੈਨੂੰ ਇਸ ਸਹਿਮਤੀ ਦਸਤਾਵੇਜ਼ ਦੀ ਕਾਪੀ ਭੇਜੀ ਗਈ।

ਪ੍ਰਤੀਭਾਗੀ ਦਾ ਨਾਮ/ਹਸਤਾਖ਼ਰ/ਅੰਗੂਠੇ ਦਾ ਨਿਸ਼ਾਨ
(ਜਾਂ ਕਾਨੂੰਨੀ ਸਰਪ੍ਰਸਤ ਜੇ ਪ੍ਰਤੀਭਾਗੀ ਅਯੋਗ ਹੋਵੇ):

__________________ (ਨਾਮ) ____________ (ਹਸਤਾਖ਼ਰ)

ਤਾਰੀਖ਼: ___________ ਸਮਾਂ: _________

ਨਿਰਪੱਖ ਗਵਾਹ ਦਾ ਨਾਮ ਅਤੇ ਹਸਤਾਖ਼ਰ (ਅਨਪੜ੍ਹ ਰੋਗੀਆਂ ਲਈ ਜਰੂਰੀ):

__________________ (ਨਾਮ) ____________ (ਹਸਤਾਖ਼ਰ)

ਤਾਰੀਖ਼: ___________ ਸਮਾਂ: _________

ਨਿਰਪੱਖ ਗਵਾਹ ਦਾ ਪਤਾ ਅਤੇ ਸੰਪਰਕ ਨੰਬਰ: _____________________

____

ਖੋਜਕਰਤਾ ਦਾ ਨਾਮ ਅਤੇ ਹਸਤਾਖ਼ਰ ਜਾਂ ਸਹਿਮਤੀ ਪ੍ਰਤੀਨਿਧੀ ਤੋਂ ਸਹਿਮਤੀ ਪ੍ਰਾਪਤ ਕਰਨਾ:

__________________ (ਨਾਮ) ____________ (ਹਸਤਾਖ਼ਰ)

ਤਾਰੀਖ਼: ___________ ਸਮਾਂ: _________

ਖੋਜਕਰਤਾ ਦਾ ਸਰਟੀਫਿਕੇਟ

ਮੈਂ ਪ੍ਰਮਾਣਿਤ ਕਰਦਾ/ਕਰਦੀ ਹਾਂ ਕਿ ਇਸ ਉਪਰੋਕਤ ਅਧਿਐਨ ਦੀ ਪ੍ਰਕਿਰਤੀ, ਉਦੇਸ਼ ਅਤੇ ਸੰਭਾਵਿਤ ਖ਼ਤਰਿਆਂ ਸਮੇਤ ਸਾਰੇ ਤੱਤਾਂ ਜਿਵੇਂ ਕਿ ਇਸ ਸਹਿਮਤੀ ਦਸਤਾਵੇਜ਼ ਵਿੱਚ ਵਰਣਿਤ ਹਨ, ਉਵੇਂ ਵੀ ਚੰਗੀ ਤਰ੍ਹਾਂ ਦੱਸੇ ਗਏ ਹਨ। ਮੇਰੇ ਨਿਰਣੇ ਵਿੱਚ, ਪ੍ਰਤੀਭਾਗੀ/ਕਾਨੂੰਨੀ ਪ੍ਰਤੀਨਿਧੀ ਇਸ ਖੋਜ ਵਿੱਚ ਭਾਗ ਲੈਣ ਲਈ ਸੂਚਿਤ ਸਹਿਮਤੀ ਦੇਣ ਦੀ ਕਾਨੂੰਨੀ ਸਮੱਰਥਾ ਰੱਖਦਾ/ਰੱਖਦੀ ਹੈ ਅਤੇ ਆਪਣੀ ਇੱਛਾ ਅਤੇ ਇਰਾਦੇ ਨਾਲ ਭਾਗ ਲੈਣ ਲਈ ਸੂਚਿਤ ਸਹਿਮਤੀ ਦੇ ਰਿਹਾ/ਰਹੀ ਹੈ।

ਖੋਜਕਰਤਾ ਦੇ ਹਸਤਾਖ਼ਰ: ____________________ ਤਾਰੀਖ਼:____________

ਖੋਜਕਰਤਾ ਦਾ ਨਾਮ: ________________________
